# ﻿Taxonomic and nomenclatural reassessment of the Iberian Peninsula’s *nomina obscura*, *Scolopendraviridipes* Dufour, 1820 and *S.chlorotes* L. Koch in Rosenhauer, 1856 (Chilopoda, Scolopendromorpha, Scolopendridae)

**DOI:** 10.3897/zookeys.1208.122126

**Published:** 2024-07-25

**Authors:** Carles Doménech

**Affiliations:** 1 Departament de Ciències Ambientals i Recursos Naturals, Universitat d’Alacant, Carretera de Sant Vicent del Raspeig s/n C.P. 03690, San Vicent del Raspeig, Alacant, Spain Universitat d’Alacant San Vicent del Raspeig, Alacant Spain

**Keywords:** Chilopoda, *chlorotes*, continental Spain, *oraniensis*, *Scolopendra*, *viridipes*

## Abstract

The taxonomic identities of the two largely neglected *Scolopendra* Linnaeus, 1758 species from continental Spain, *S.viridipes* Dufour, 1820 and *S.chlorotes* L. Koch in Rosenhauer, 1856, are examined in this paper. After efforts in locating both species’ type series in eight European institutions, the specimens are considered to be lost. Consequently, the identifications of both taxa were approximated by collating their descriptions with the morphology of all other sympatric Scolopendromorpha. Then, compatible topotypes for both species were collected, and among these a neotype for each taxon were selected and compared with the type series of their respective closest relatives. Finally, both *S.viridipes* and *S.chlorotes* are proposed to be conspecific with *S.oraniensis* Lucas, 1846. Therefore, the name *S.viridipes* is here established as (senior) **syn. nov.** and **nomen oblitum** of *S.oraniensis*, *S.oraniensis* is declared as **nomen protectum**, and *S.chlorotes* (junior) **syn. nov.** is reallocated to *S.oraniensis*. Moreover, the specimens making up the type series of *S.oraniensis* are also indicated and redescribed, the genitalia are illustrated for the first time, and its specific epithet is briefly reviewed, remaining unaltered in respect of its original spelling.

## ﻿Introduction

The class Chilopoda Latreille, 1817 is a group of venomous terrestrial predators that play an important ecological role in the warm and temperate ecosystems. Taxonomically, this group of arthropods encompasses approximately 3,100 species distributed in five orders (Minelli 2011). The best known of all of them is the order Scolopendromorpha, which currently counts a total of six accepted families subdivided into 37 genera and subgenera (Schileyko et al. 2020).

**Figure 1.**
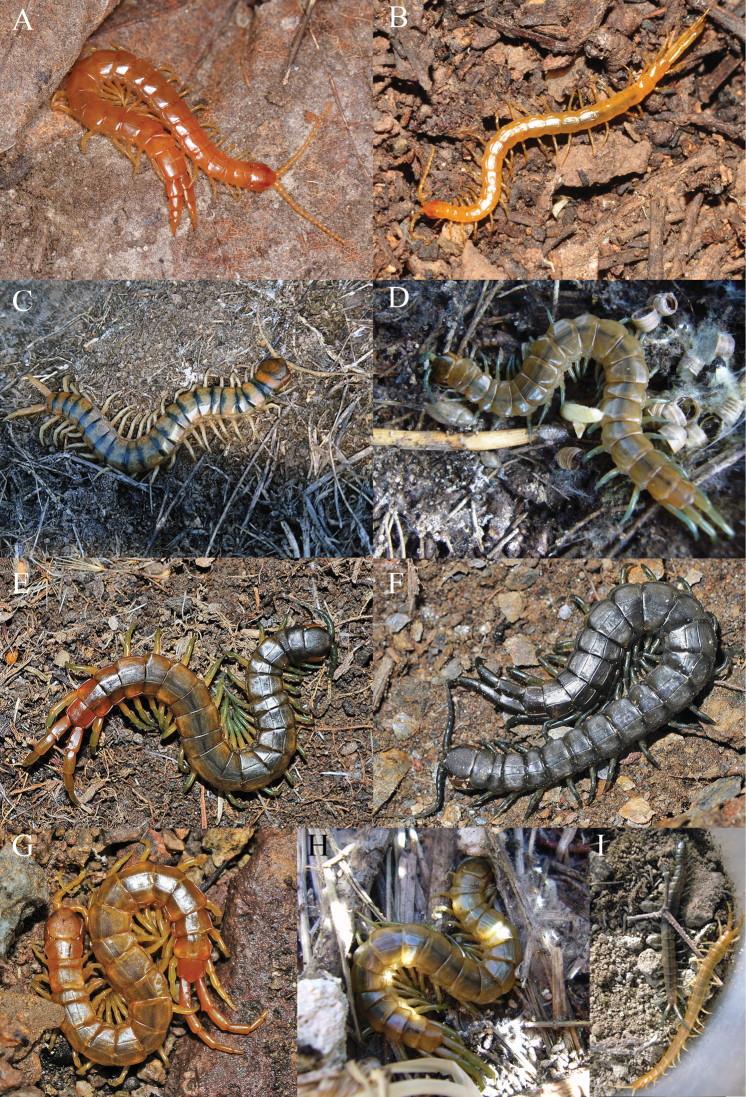
Representatives of Scolopendromorpha from the Iberian Peninsula (uncollected) **A** family Plutoniumidae Bollman, 1893: *Theatopserytrocephalus* (C. L. Koch, 1847), Seoane do Caurel, Lugo (credit: J. Tizón) **B** family Cryptopidae Kohlrausch, 1881: *Cryptops* sp. Leach, 1814, Villena, Alacant (credit: D. Molina) **C–I** family Scolopendridae Leach, 1814 **C***Scolopendracingulata* Latreille, 1829, Benilloba, Alacant (credit: M. Huesca and CD) and **D–I***S.oraniensis* Lucas, 1840. Observe that in this species the colour variants can occur sympatrically **D, H, I** Benilloba, Alacant and **E–G** Almeria Province, Andalucia (credit: F. Rodriguez Luque).

In peninsular Spain, the order Scolopendromorpha is represented by three of those six families. One of them, Plutoniumidae Bollman, 1893 (Fig. 1A), is composed of two extant genera: the monotypic *Plutonium* Cavanna, 1881 and *Theatops* Newport, 1844, the latter only represented by a single species. The second family, and the most diverse one in the peninsula, is the monotypic family Cryptopidae Kohlrausch, 1881 (Fig. 1B). This taxon includes two *Cryptops* Leach, 1814 subgenera, and a total of eight species. The third is the family Scolopendridae Leach, 1814 (Fig. 1C–I), which is represented by one genus, *Scolopendra* Linnaeus, 1758, and two reported taxa, *S.cingulata* Latreille, 1829 and *S.oraniensis* Lucas, 1846 (Vadell 2013; Giribet 2015; Bonato et al. 2017b).

With the general exception of the north and northwest, these two *Scolopendra* species are widely and almost sympatrically distributed in continental Spain (Serra 1983; García-Ruiz 2018; Cabanillas 2019; Cabanillas et al. 2019; Cabanillas and Parejo-Pulido 2019; Cabanillas and García-Febrero 2020; Doménech et al. 2023). *Scolopendracingulata* is the longest, reaching up to 80–90 mm (García-Ruiz 2007). Even as a juvenile, this species has a characteristic colouration of yellow tergites posteriorly pigmented by a blackish horizontal band—the “*cingulum*”; from Latin for belt or cord—that distinguishes it by sight from its local Iberian relatives (Fig. 1C). The other species, *S.oraniensis*, still often incorrectly referred to as a *S.canidens* Newport, 1844 subspecies (Würmli 1980; Serra 1983; Carballo and Daza 1991; García-Ruiz 1993, 2018), is a smaller centipede which only reaches up to 65 mm. Its colouration is the most variable within the Iberian Scolopendromorpha, with legs and antennae from pale yellow to greenish, blueish, or reddish, and the dorsal habitus with various combinations of colour from cream to dark brown that can vary even sympatrically (Fig. 1D–I).

**Figure 2.**
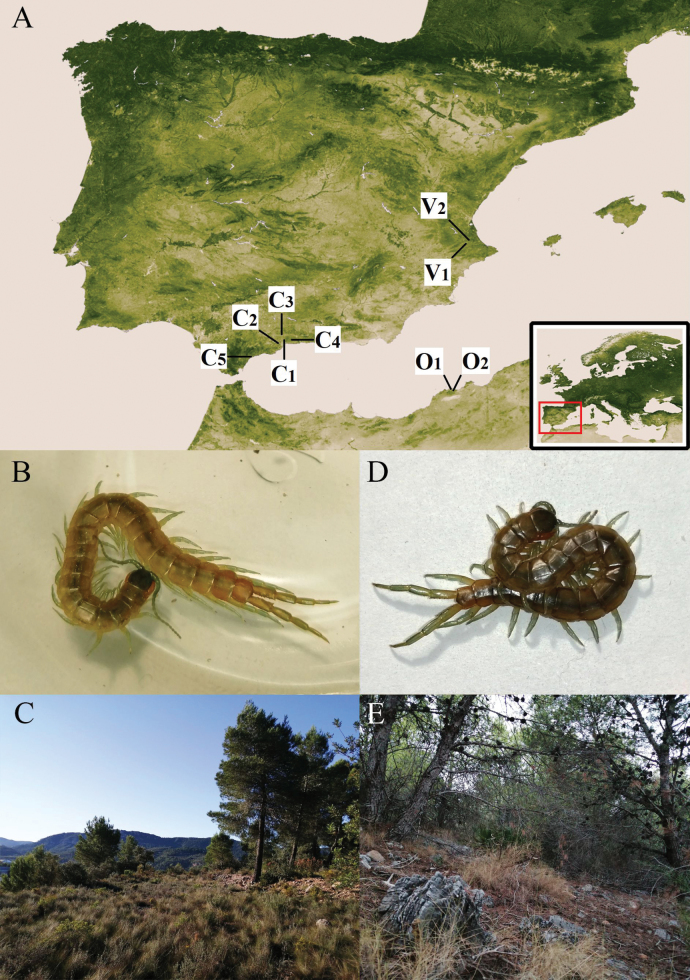
**A** topotype collection localities in the Iberian Peninsula (southwestern Europe): *S.viridipes* topotypes (= *S.oraniensis*); V_1_ = Moixent and V_2_ = Xàtiva (previously San Felipe), Valencia province, Valencian Country/Community, Spain and *S.chlorotes* topotypes (= *S.oraniensis*); C_1_ = Málaga municipality, C_2_ = Alahurín de la Torre, C_3_ = Casabermeja, C_4_= Totalán and C_5_ = Estepona, Málaga province, Andalucía, Spain. The “O” points *S.oraniensis* type localities, O_1_ = Santon’s mountains [corrected from “Sauton” (sic.); Lucas 1846] and O_2_ = “Between Oran and Mers-el-Kebir”, Oran province (Wilayah), Algeria **B** “*S.viridipes*” topotype 2, female, (CEUAMr22) from **C** Moixent, Alacant **D** “*S.chlorotes*” topotype 6, female, (CEUAMr30) from **E** Alahurín de la Torre, Málaga.

However, besides *S.semipedalis* Dufour, 1820 (= *Himantariumgabrielis* (Linnaeus, 1767), see Bonato and Minelli 2014) and *S.venefica* L. Koch in Rosenhauer, 1856 (= *S.cingulata*, see Attems 1930), two additional and until now largely forgotten species from the Iberian Peninsula were included in the genus *Scolopendra*. The first one is *S.viridipes* Dufour, 1820, a lapidicolous and small-sized centipede described from an unspecified place in the “Mountains of Kingdom of Valencia” (east of continental Spain) (Fig. 2A–C). Aside from the description and subsequent transcription (Lucas 1840), the literature on this species is mostly limited to species lists (Gervais 1837; Lamarck 1838, 1839; Lucas 1840; Laboulbène 1865). However, based only on the literature, some authors hypothesised about its taxonomic relationships and actual identity: Brandt (1840) and Ranzani (1841) considered that *S.clavipes* C. L. Koch, 1836 [not 1847, see Doménech and Nagel 2022] could actually be a junior synonym of this species. Walckenaer and Gervais (1844) concluded that *S.viridipes* was too poorly described for proper recognition, while Pirotta (1878a) questioned whether his own recently described species, *S.doriae* Pirotta, 1878b (= *S.cingulata*), was simply another synonym of *S.viridipes*. Soon after, Kraepelin (1903) listed *S.viridipes* [as ?*S.viridipes* Dufour, 1860 (sic.)] as a possible synonym of *S.oraniensis*, and finally, Attems (1930) included *S.viridipes* Dufour, 1822 (sic.) in the list of “non-recognizable taxa”.

The second species in this underappreciated group is *S.chlorotes* L. Koch in Rosenhauer, 1856, another small, but much better described taxon from “near Málaga”, Andalusia (south of continental Spain) (Fig. 2A, D, E). Only four references to this species were found that include its original description, a transcription (Rosenhauer 1856) and two taxonomic mentions: *S.chlorotes* was also listed by Kraepelin (1903) as a possible synonym of *S.oraniensis*, while Attems (1930) declared *S.chlorotes* as an unrecognisable species.

After more than nine decades of bibliographic silence, the uncertain taxonomic and nomenclatural identities of the two enigmatic species *S.viridipes* and *S.chlorotes* are now evaluated, a specific diagnosis for each species is given, and the nomenclatural situation for these taxa is finally clarified.

## ﻿Materials and methods

The depositories of the types series of *Scolopendraviridipes*, *S.chlorotes*, and *S.oraniensis* were scrutinised following the texts of Dufour (1820), Lucas (1846), L. Koch (1856), Kraepelin (1904), Rühm (1925), Moritz and Fischer (1979) and Hessel (2000). The additional data from the depositories were obtained from https://www.idigbio.org, https://bionomia.net, and http://sdei.senckenberg.de/biographies websites (last accessed on 21 Jan. 2022). Depository and other institutions abbreviations are as follows:

**ASU**Altai State University, Altai Krai, Russia

**CEUA**Colección Entomológica de la Universidad de Alicante San Vicent del Raspeig, Alacant, Spain

**CLD** Cercle Léon Dufour. Saint Severe, Nouvelle-Aquitaine, France

**FAU** Friedrich-Alexander Universität, Erlangen-Nürnberg, Erlangen, Germany

**MNCN**Museo Nacional de Ciencias Naturales, Madrid, Spain

**MNHN**Muséum national d’Histoire Naturelle, Paris, France

**MZS**Musée Zoologique, Strasbourg, France

**NHMB**Naturhistorisches Museum Bern, Switzerland

**NHMN** Naturhistorisches Museum Nürnberg, Nürnberg, Germany

**NHMUK**Natural History Museum, London, UK

**SAE** Sociedad Andaluza de Entomología, Dos Hermanas, Sevilla, Spain

**SLB** Société Linnéenne de Bordeaux, Bourdeaux, France

**UPV** Universitat Politècnica de València, València, Spain

**ZMB**Museum für Naturkunde, Berlin, Germany

Topotypes locations of *Scolopendraviridipes* are based on information given in Dufour (1888), Hessel (2000), and Ferrández (2020). From these, only the inland mountainous localities with Dufour’s (1820) description of colour-matching specimens were chosen (Fig. 2A–C). *Scolopendrachlorotes* topotypes were collected in the surrounding areas of Málaga City and in the municipality’s surroundings following L. Koch (1856) (Fig. 2A, D, E). Specimens from these areas, representing the nominal species *S.viridipes* and *S.chlorotes*, were manually collected and soaked in 50% ethanol for 15 min. For preservation of the samples, individual containers with 70% ethanol were used. Morphological features were checked and photographed under a Leica M205C stereomicroscope connected to a montage imaging system, Leica DFC450, operated under the Cell’D program at the Universidad de Alicante (**UA**), Spain. Measurements were made with a Monzana® Digital Vernier Caliper. All specimens were collected following the indications in permits granted by the Generalitat Valenciana (Exp. 093/20 FAU20_006; grant date: 17 Feb. 2020) and Junta de Andalucía (N/Ref.: SGYB/DBP; grant date: 15 May 2021). Throughout the text, the term topotype [without quotation marks] will refer to specimens collected at the type locality of the original descriptions. The term “topotype”, with quotes, indicates that they have lost this original topotype designation in favour of the one of the neotype (ICZN 1999: Art. 76.1.1, 76.1.6).

The type series of *Scolopendraoraniensis* in the MNHN was identified following Lucas (1840) and Kraepelin (1904), as well as the label information found in the jars. All specimens were examined using a Wild Heerbrugg M3C stereomicroscope at the MNHN. Illustrations of morphological features were achieved using a Canon digital camera model EOS 6D with a MP-E 65 mm lens operated with Helicon Remote v. 3.9.1.W system. Morphological abbreviations used in text are as follows:

### ﻿General morphology

**AP** apical spines

**DM** dorso-median spines

**LS** lateral spines

**M** median spines

**S**, **SS** sternite, sternites

**SAP** subapical spines

**SP** prefemoral process spines

**T**, **TT** tergite, tergites

**UL** ultimate legs

**ULBS** ultimate leg-bearing segment

**V** ventral spines

**VL** ventro-lateral spines

**VM** ventro-median spines

**Genital region**

**AV** anal valve

**LA** lamina adanalis

**LS** lamina subanalis

**SGS I** sternite of genital segment 1

**SGS II** sternite of genital segment 2

The species identifications and differential diagnosis in Tables 1, 2 are based on the previous works of Dufour (1820), Lucas (1846), L. Koch (1856), Cavanna (1881), Kraepelin (1903), Ribaut (1915), Attems (1930), Verhoeff (1931, 1934), Machado (1953), Matic and Darabantu (1968), Würmli (1980), Serra (1981, 1983), Iorio and Geoffroy (2006, 2008), Di et al. (2010), Lewis (2010, 2011), Vadell (2013), Voigtländer and Reip (2013), Giribet (2015), Bonato et al. (2017b), and Schileyko et al. (2020).

**Table 1.** The original descriptive morphology of *Scolopendraviridipes* in comparison to other Scolopendromorpha species described in Peninsular Spain. **Bold letters** indicate compatible features in other species. * indicates common to the entire Scolopendromorpha, interpretable, or poorly differentiated feature. Nr^pp^= Not reported, presence possible.

	*Scolopendraviridipes* Dufour, 1820	*S.cingulata* Latreille, 1829	*S.oraniensis* Lucas, 1846	Cryptops (Cryptops) anomalans Newport, 1844	C. (C.) hispanus Brölemann, 1920	C. (C.) hortensis*s.s* (Donovan, 1810)	C. (C.) lobatus Verhoeff, 1931	C. (C.) parisi*s.s* Brölemann, 1920	C. (C.) trisulcatus Brölemann, 1902	C. (Trygonocryptops) longicornis (Ribaut, 1915)	C. (T.) similis Machado, 1953	*Plutoniumzwierleini* Cavanna, 1881	*Theatopserythrocephalus* (C. L. Koch, 1847)
**Presence in Valencianish Kingdom**	Present	**Present**	**Present**	Nr^pp^	**Present**	Nr^pp^	Absent	Nr^pp^	Nr^pp^	Absent	Nr^pp^	Absent	**Present**
**Ecology**	Edaphic epigean (Lapidicola)	**Edaphic epigean**, troglophile	**Edaphic epigean**, troglophile	**Edaphic epigean**	**Edaphic epigean**, troglophile*	**Edaphic epigean**, troglophile*	**Edaphic epigean**	**Edaphic epigean**	**Edaphic epigean**	Troglobiotic	**Edaphic epigean**	**Edaphic, hipogean**, troglophile	**Edaphic epigean**, troglophile
**Colouration**	Marked pale, with antenna and legs greenish	Tergites yellowish ended with black distal edge, legs and antenna yellow to orange	**Pale cream to dark green or brown with antenna and legs yellowish, greenish, bluish or reddish**	Pale yellow to reddish	Pale yellow to reddish	Reddish, sometimes pale yellow	Pale yellow to reddish	Reddish	Pale yellow	Pale yellow	Pale yellow	Pale reddish, legs and antennae usually yellowish	Pale yellow to reddish, cephalic plate and UL usually darker
**Length (in mm)**	40.6 (18 lignes)	100-155	**40-65**	**20-40**	16-30	12-30	10-13.5	14-21	15-35	**27-38**	30	**50-80**	**20-45**
Antennal articles’ number*	More than 15*	**18**	**18-19**	**17**	**17**	**17**	**17**	**17**	**17**	**17**	**17**	**17**	**17**
Antennae end in a setaceous point*	Yes*	**Yes**	**Yes**	**Yes**	**Yes**	**Yes**	**Yes**	**Yes**	**Yes**	**Yes**	**Yes**	**Yes**	**Yes**
Headplate shape*	Small* and oval	**Oval**	**Oval**	**Oval**	**Oval**	**Oval**	**Oval**	**Oval**	**Oval**	**Oval**	**Oval**	**Oval**	**Oval**
**Shape of the distal palp’s article** (article 3 in second maxillae’s telopodite) *	Dilated and round *	**Subcylindrical**	**Subcylindrical**	**Subcylindrical**	**Subcylindrical**	**Subcylindrical**	**Subcylindrical**	**Subcylindrical**	**Subcylindrical**	**Subcylindrical**	**Subcylindrical**	**Subcylindrical**	**Subcylindrical**
1^st^, 2^nd^ and last tergites different in shape respect the others*	Yes*	**Yes**, smaller in comparison	**Yes**, smaller in comparison	**Yes**, smaller in comparison	**Yes**, smaller in comparison	**Yes**, smaller in comparison	**Yes**, smaller in comparison	**Yes**, smaller in comparison	**Yes**, smaller in comparison	**Yes**, smaller in comparison	**Yes**, smaller in comparison	**Yes**, 1^st^ and 2^nd^ smaller in comparison, the 21^st^ much bigger	**Yes**, 1^st^ and 2^nd^ smaller in comparison, the 21^st^ much bigger
**Ultimate legs length respect the locomotory legs**	Longer than locomotor ones	**Longer than locomotor ones**	**Longer than locomotor ones**	**Longer than locomotor ones**	**Longer than locomotor ones**	**Longer than locomotor ones**	**Longer than locomotor ones**	**Longer than locomotor ones**	**Longer than locomotor ones**	**Much longer than locomotor ones**	**Longer than locomotor ones**	Similar in length to locomotor ones, shape markedly thicker	Similar in length to locomotor ones, shape markedly thicker

**Table 2.** The original descriptive morphology of *Scolopendrachlorotes* in comparison to other Scolopendromorpha species described in Peninsular Spain. **Bold letters** indicate compatible features in other species. * indicates common to the entire Scolopendromorpha, interpretable, or poorly differentiated feature. Nr^pp^= Not reported, presence possible. NA= Information not available.

	*Scolopendrachlorotes* L. Koch in Rosenhauer, 1856	*S.cingulata* Latreille, 1829	*S.oraniensis* Lucas, 1846	Cryptops (Cryptops) anomalans Newport, 1844	C. (C.) hispanus Brölemann, 1920	C. (C.) hortensis s. s. (Donovan, 1810)	C. (C.) lobatus Verhoeff, 1931	C. (C.) parisi s. s Brölemann, 1920	C. (C.) trisulcatus Brölemann, 1902	C. (Trygonocryptops) longicornis (Ribaut, 1915)	C. (T.) similis Machado, 1953	*Plutoniumzwierleini* Cavanna, 1881	*Theatopserythrocephalus* (C. L. Koch, 1847)
**Presence in Malaga**	**Present**	**Present**	**Present**	Nr^pp^	**Present**	Nr^pp^	Absent	Nr^pp^	Nr^pp^	**Present**	**Present**	**Present**	**Present**
**Length (in mm)**	**42.22** (20 lignes)	100-155	**40-65**	**20-40**	16-30	12-30	10-13.5	14-21	**15-35**	**27-38**	30	**50-80**	**20-45**
**Colouration**	**Head-plate, antenna and tergites, brownish-green, maxillipeds, last tergite and UL reddish brown, and legs yellowish proximally and greenish distally**	Tergites yellowish ended with black distal edge, legs and antenna yellow to orange	**Pale cream to dark green or brown with antenna and legs yellowish, greenish, bluish or reddish**	Pale yellow to reddish	Pale yellow to reddish	Reddish, sometimes pale yellow	Pale yellow to reddish	Reddish	Pale yellow	Pale yellow	Pale yellow	Pale reddish, legs and antennae usually yellowish	Pale yellow to reddish, cephalic plate and UL usually darker
Headplate shape*	**Oval**	**Oval**	**Oval**	**Oval**	**Oval**	**Oval**	**Oval**	**Oval**	**Oval**	**Oval**	**Oval**	**Oval**	**Oval**
**Tooth-plate on coxosternum**	**Present**	**Present**	**Present**	Absent	Absent	Absent	Absent	Absent	Absent	Absent	Absent	**Present**	**Present**
Tergites shape*	**Tergite sides straight, TT1-3 and 20 rounded anteriorly**	**Quadrangular TT1-2 and T21 slightly smaller**	**Quadrangular TT1-2 and T21 slightly smaller**	**Quadrangular TT1-2 and T21 slightly smaller**	**Quadrangular TT1-2 and T21 slightly smaller**	**Quadrangular TT1-2 and T21 slightly smaller**	**Quadrangular TT1-2 and T21 slightly smaller**	**Quadrangular TT1-2 and T21 slightly smaller**	**Quadrangular TT1-2 and T21 slightly smaller**	**Quadrangular TT1-2 and T21 slightly smaller**	**Quadrangular TT1-2 and T21 slightly smaller**	**Quadrangular TT1-2 slightly smaller**, Segment 21 longitudinal enlarged	**Quadrangular TT1-2 slightly smaller**, Segment 21 longitudinal enlarged
**TT’s paramedian sutures**	**TT2-20**	**TT2/3-20**	**TT2-20**	**TT2-20**	**Sutures weak, apparently present since T2**	**TT3, 4 or 5-20**	**TT2-20**	**TT2/3-20**	**TT2-20**	**TT2, 3 or 4-20**	**TT2-20**	**TT2-20**	**TT2-20**
Tergites margination*	**TT14-21**	**TT7/12-21**	**Complete from TT19-21**	**TT3-19**	NA	TT3, 4 or 5-20*	NA	**NA**	**TT3-20**	**TT4-21**	**TT3-20**	T21	T21
**Median longitudinal suture on the T21**	**Present**	Absent	**Present**	**Presence observed variable**; T21 with a depression	NA	NA, probably absent; T21 with a depression	NA, probably absent	Absent	**Present**	Absent	NA	**Present**	**Present**
**Paramedian sutures on sternites**	**Longitudinal, absent in S 21**	**SS2-20**	**SS2-20**	Cruciform sutures	Cruciform sutures	Cruciform sutures	Cruciform sutures	Cruciform sutures	Cruciform sutures	Trigonal sutures	Trigonal sutures	Single medial longitudinal suture	Single medial longitudinal suture
**UL’s prefemoral process**	**Short, with two blunt spines at the extreme**	**Short, with 3-5 spines**	**Short, with 2 or 3, rarely up to 6 spines**	Absent	Absent (femur and tibia with 4 distal processes)	Absent	Absent	Absent	Present, with a single spine (femur with a distal process)	Absent	Present, with a single spine (femur with a distal process, tibia with distal two processes)	Process absent	Process absent
**UL’s spinous prefemoral formula**	**V: 19 spines arranged in for rows, M:7 spines**	VL:0; V: 2; VM: 0; M: (1)2; DM: (1)2	**VL:3-5; V: 3-6; VM: 2-6; M: 4-8; DM:2-4**	Prefemur with setae, spines absent,7-10 saw teeth on tibia and 3-5 on tarsus 1	Prefemur with setae, spines absent, 8 saw teeth on tibia and 4 on tarsus 1	Prefemur with setae, spines absent, 5-8(9) saw teeth on tibia and 2-4 on tarsus 1	Prefemur with setae, spines absent, 6-10 saw teeth on tibia and 4-6 on tarsus 1	Prefemur with setae, spines absent, 6-12 saw teeth on tibia and 4-8 on tarsus 1	Prefemur with setae, spines absent, 13 saw teeth on tibia and 4 on tarsus 1	Prefemur with setae, spines absent, 12-13 saw teeth on tibia and 5 on tarsus 1	Prefemur with setae, spines absent, 10 saw teeth on tibia and 3 on tarsus 1	Spines absent	With one ventromedial prefemoral and one femoral spine

The distribution of *Scolopendra* species colour variants were defined using the **BV** (Biodiversidad Virtual 2024) website with only the confirmed identifications of D. Cabanillas (BV’s expert) and the author together with the texts of Voigtländer and Reip (2013), Cabanillas and García-Febrero (2020), and field observations. Standardised nomenclature for centipede morphology was applied following Bonato et al. (2010). Sex determination and genitalia descriptions have been performed using Demange and Richard (1969), Iorio (2003), Iorio and Geoffroy (2006), and Siriwut et al. (2016). Nomenclatural acts, including the neotypes designations, were made according to the rules of the Arts. 23.2, 23.3, 23.9.1.1, 23.9.1.2, 23.9.2, 23.9.6, 32.5, 58, 58.15, 72.4.1.1, 73.1.2, 74.7, 75.1–75.3, and 76 in the fourth edition of the International Code of Zoological Nomenclature (ICZN 1999).

Background removal in illustrations, contrast adjustments, map configuration, and their respective clarifying notes were performed with Adobe Photoshop CS6 software®. A base map was obtained from the National Oceanic and Atmospheric Administration, National Weather Service (NOAA/NWS 2022) website.

### ﻿Material examined

**Figure 3.**
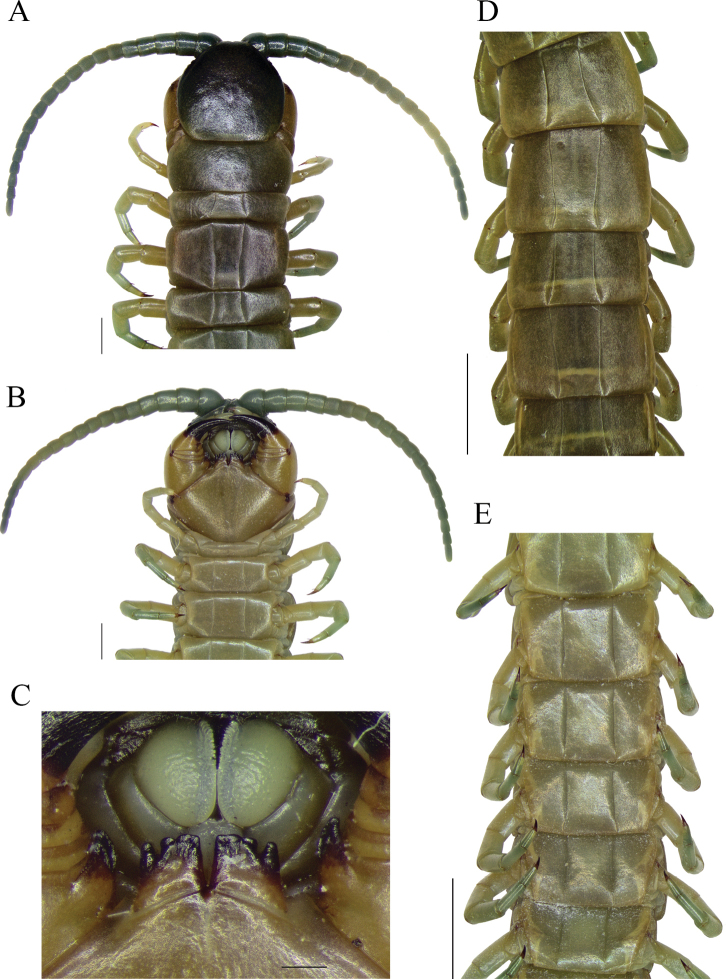
*Scolopendraviridipes* neotype (= *S.oraniensis*); male; (“topotype 1", CEUAMr21) **A** cephalic plate, antennae, and TT 1–5 **B** forcipular segment and sternites 1–3 **C** tooth plates **D** tergites 13–18 **E** sternites 10–15. Scale bars: 0.2 mm (**C**); 1 mm (**A, B**); 2 mm (**D, E**).

**Figure 4.**
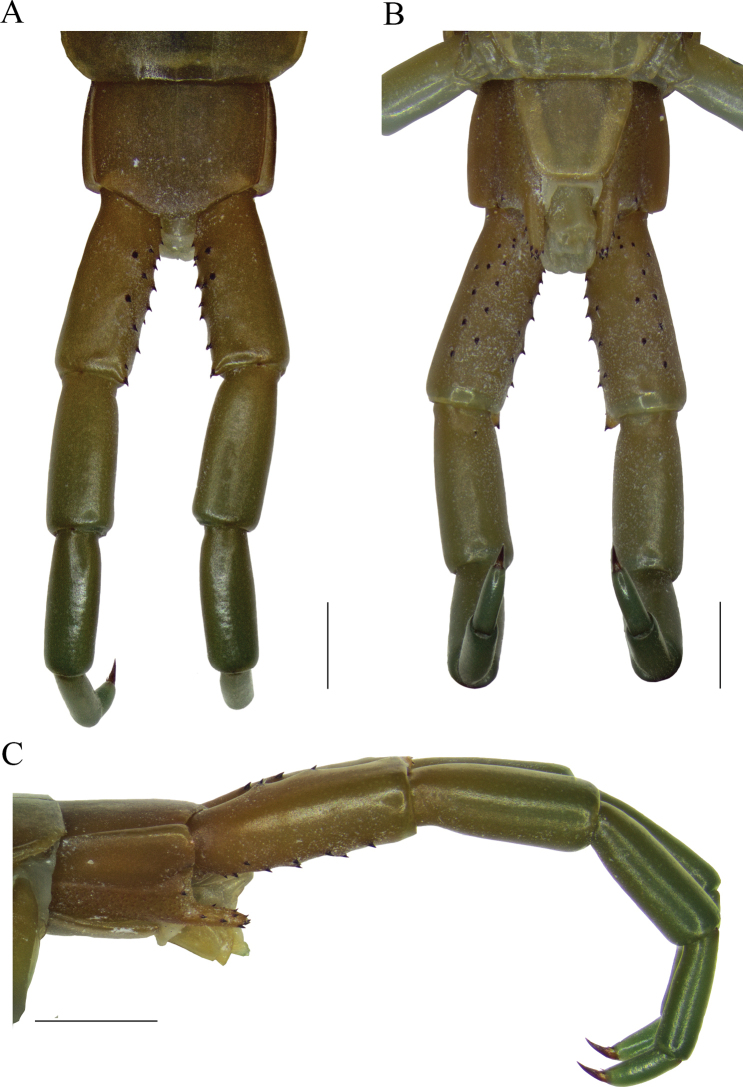
*Scolopendraviridipes* neotype (= *S.oraniensis*); male; (“topotype 1", CEUAMr21) **A**ULBS and UL’s dorsal view **B**ULBS’s and UL’s ventral view **C**ULBS’s, coxopleuron, and UL’s ventral lateral view. Scale bars: 1 mm (**A, B**); 2 mm (**C**).

**Figure 5.**
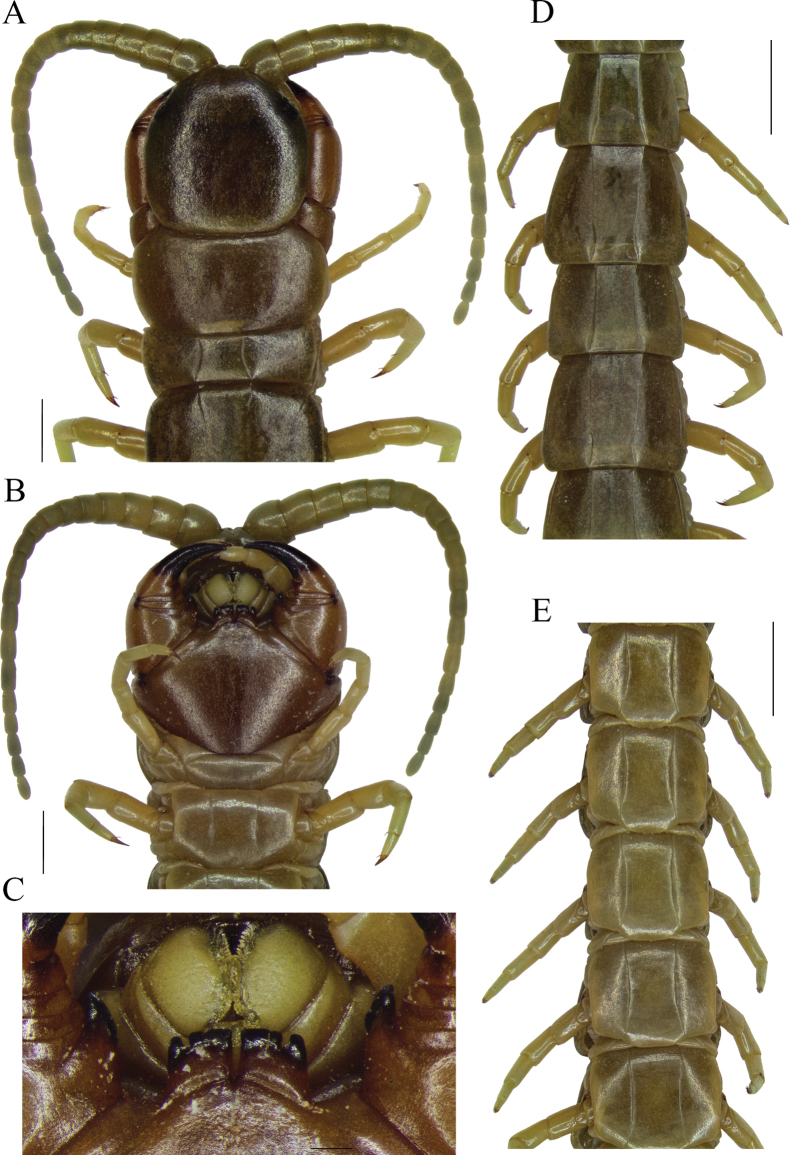
*Scolopendrachlorotes* neotype (= *S.oraniensis*); male; (“topotype 5", CEUAMr29) **A** cephalic plate, antennae, and TT1–3 **B** forcipular segment and sternites 1, 2 **C** tooth plates **D** tergites 10–14 **E** sternites 8–12. Scale bars: 0.2 mm (**C**); 1 mm (**A, B**); 2 mm (**D, E**).

**Figure 6.**
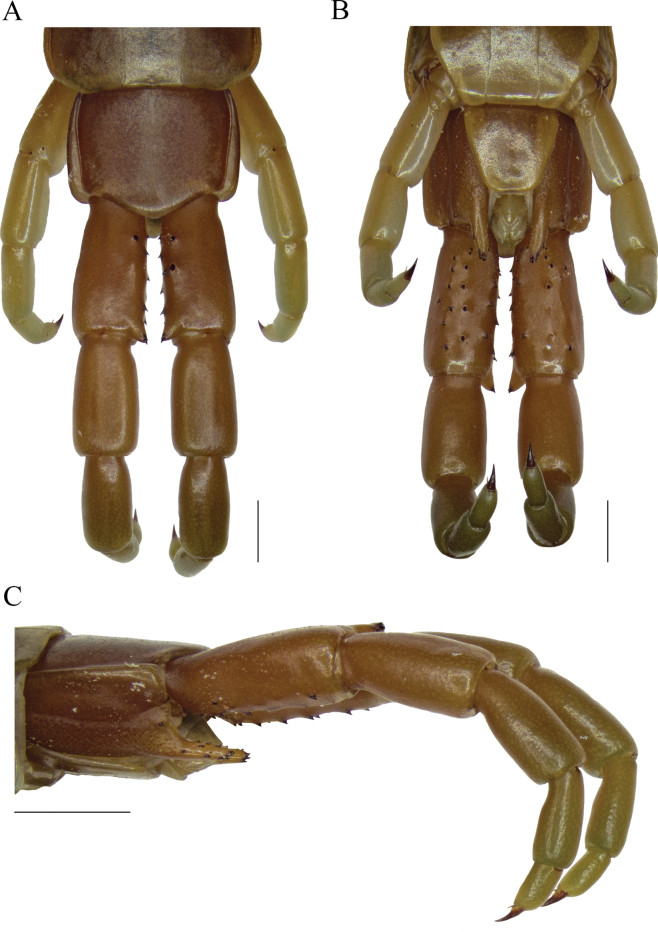
*Scolopendrachlorotes* neotype (= *S.oraniensis*); male; (“topotype 5", CEUAMr29) **A**ULBS’s and UL’s dorsal view **B**ULBS’s and UL’s ventral view **C**ULBS’s, coxopleuron, and UL’s ventral lateral view. Scale bars: 1 mm (**A, B**); 2 mm (**C**).

**CEUA**: **Spain** • 2 ♂ and 2♀ adults *S.viridipes* “topotypes” (CEUAMr21–25): Valencia Province: Moixent; Embassament del Bosquet, (38°51'21.7"N, 0°44'36.7"W 380 m a.s.l.) (Fig. 2B, C), 1 ♂ (Neotype, CEUAMr005, Figs 3, 4, Table 3, Suppl. material 1: file 1) and 1♀; Coll. 26 Sep. 2020 and 1 ♂ and 1♀ Xàtiva, Pujada Bixquert (38°58'56.4"N, 0°30'25.7"W, 115 m a.s.l.); Coll. 3 Oct. 2020, Lapidicolous; C. Doménech leg. (Figs 2A–C, 3, 4, 9A, C; Table 3; Suppl. material 1: file 1). • 4 ♂, 8♀ (1 subadult) and 3 unsexed (1 subadult) individuals *S.chlorotes* “topotypes” (CEUAMr25 to 39): Málaga province, 4♀ (1 subadult), Málaga Municipality, next to park Cementerio (36°43'27.7'’N, 4°31'10.8'’W 71 m a.s.l.); 1 ♂ (Neotype, CEUAMr29, Figs 5, 6; Table 3; Suppl. material 1: file 3) and 1♀, Alahurín de la Torre, Carretera de Coín, (36°39'37'’N, 4°31'0.4'’W 104 m a.s.l.) (Fig. 2D, E); 1 ♂, 2♀ and 1 unsexed adults; Casabermeja, el Chorro, (36°59'14.9'’N, 4°25'52.5'’W 649 m a.s.l.); 2 ♂ and 1 unsexed subadult, Totalán, Arroyo de Sixto (36°45'04.1'’N, 4°18'41'’W 154 m a.s.l.); Coll. 18 and 19 Nov. 2021; C. Doménech Leg.; 1♀ and 1 unsexed, Estepona (36°25'59"N, 5°07'59"W 115m a.s.l.), Coll. 17 Jun. 2021. R. Perez Ríos leg. Lapidicolous (Figs 2A, D, E, 5, 6, 9B, D; Table 3; Suppl. material 1: file 3).

**Table 3.** *Scolopendraoraniensis* type series morphological comparison with *S.viridipes*’ and *S.chlorotes*’ topotypes (= *S.oraniensis*; composite data from Supplementary tables 1 and 2). AP, apical spines; SAP, subapical spines; DS, dorsal spines; LS, lateral spines; VL, ventro-lateral spines; V, ventral spines; VM, ventro-median spines; M, median spines; DM, dorso-median spiness; SP, prefemoral process spines; UL; ultimate legs, ULBS, ultimate leg-bearing segment; T, tergite; TT, tergites, S, sternite; SS, sternites, RG, Retracted genitalia; * No visible (cephalic plate flexed over firsts SS), interpretable or damaged.

	*Scolopendraoraniensis* (type series)							*S.viridipes* (CEUAMr21-25)	*S.chlorotes* (CEUAMr26-40)
	Syntype “1"	Syntype “2"	Syntype “3"	Syntype “4"	Syntype “5"	Syntype “6"	Syntype “7"		
**Body length in mm**	64	60	58	52	42	41	41	35-40	26-60
**Sex**	RG	RG	RG, probably female	RG, probably female	RG	RG	RG	–	–
Antenna reaching to tergite*	T2*	T2*	T2*	T2*	T2*	T2*	T2*	T3	T3
**Number of antennal articles**	18/19	18/19	19/18	19/19	19/18	19/19	18/19	17-20	17-20
**Number of proximal glabrous articles**	5½	5½	5½	5½	5½	5½	5½	5	5-5½
**Teeth on tooth plate**	4+4	*	4+4	4+4	3+3	4+4	4+4	4+4	3+3; 4+4
**Teeth on forcipular trochanteroprefemoral processes Total (apical /medial)**	3(1/2) - 3(1/2)	*	3 (1/2) - 3 (1/2)	2 (1/1) - 3 (1/2)	2 (1/1) - 2 (1/1)	3(1/2) - 3(1/2)	3(1/2) - 3(1/2)	3(1/2)	2(1/1); 3(1/2)
**Tergite paramedian sutures**	TT2-20	TT2-20	TT2-20	TT2-20	TT2-20	TT2-20	TT2-20	TT2-20	TT2-20
**Longitudinal suture on T21**	Present	Present	Present	Present	Present	Present	Present	Present	Present
**First tergite with complete margination**	19	19	19	19	19	19	19	17-19	14-18
**Paramedian sutures on sternites**	SS2-20	SS2-20	SS2-20	SS2-20	SS2-20	SS2-20	SS2-20	SS2-20	SS2-20
**Spines in coxopleuron (Left/Right)**	1/1	1/1	1/1	1/1	1/1	1/1	1/1	1/1	1/1
**Spines in coxopleural process (Left/Right)**	AP-SAP: 4/5 DS: 2/1 LS: 4/4 Total:10/10	AP-SAP: 5/4 DS: 2/1 LS: 4/4 Total:11/9	AP-SAP: 6/5 DS: 1/2 LS: 6/6 Total:13/13	AP-SAP: 5/6 DS: 3/2 LS: 6/4 Total:14/12	AP-SAP: 4/5 DS: 2/2 LS: 4/6 Total:10/12	AP-SAP: 6/5 DS: 3/2 LS: 3/4 Total:12/11	AP-SAP: 5/5 DS: 3/3 LS: 4/5 Total:12/13	AP-SAP: 4-8 DS: 1-3 LS: 2-3 Total: 7-14	AP-SAP: 4-7 DS: 0-4 LS: 1-4 Total: 5-15
**Tarsal spurs on leg 1 (Left/Right)**	2/2	2/2	2/1	2/2	2/2	2/2	2/2	0-2	1-2
**Legs with one tarsal spur**	2-19 (Left 1-18)	2-19	2-19	2-19 (Right 1-18)	2-19	2-19	2-19	2-19	2-18 or 19
**Ultimate legs prefemoral spinulation and spines in prefemoral process (Left/Right)**	VL:4/3 V: 6/6 VM: 4/4; M: 4/4; DM:2/2; SP:2/2; Total: 22/21	VL: 3/5; V: 3/5; VM: 5/6; M: 8/6; DM:4/4; SP:3/2; Total: 26/28	VL:1/3; V: 3/2; VM: 4/4; M: 5/5; DM:2/2; SP:3/4; Total: 18/20	VL:5/5; V: 5/4; VM: 3/2; M: 5/6; DM:2/2; SP:3/3; Total: 23/22	VL:4/5; V: 4/6; VM: 5/4; M: 4/6; DM:3/4; SP: 2/3; Total: 22/21	VL:6/5; V: 5/5; VM: 3/5; M: 3/6; DM:2/2; SP:2/2; Total: 21/25	VL: 3/5; V: 3/5; VM: 5/6; M: 6/6; DM:2/3; SP:3/2; Total: 22/27	VL: 3-7; V: 4-6; VM: 3-7; M: 5-8; DM: 2; SP: 2-3; Total: 19-33	VL: 2-10; V: 4-10; VM: 2-8; M: 2-10; DM: 1-7; SP: 0-5; Total: 11-50

**Figure 7.**
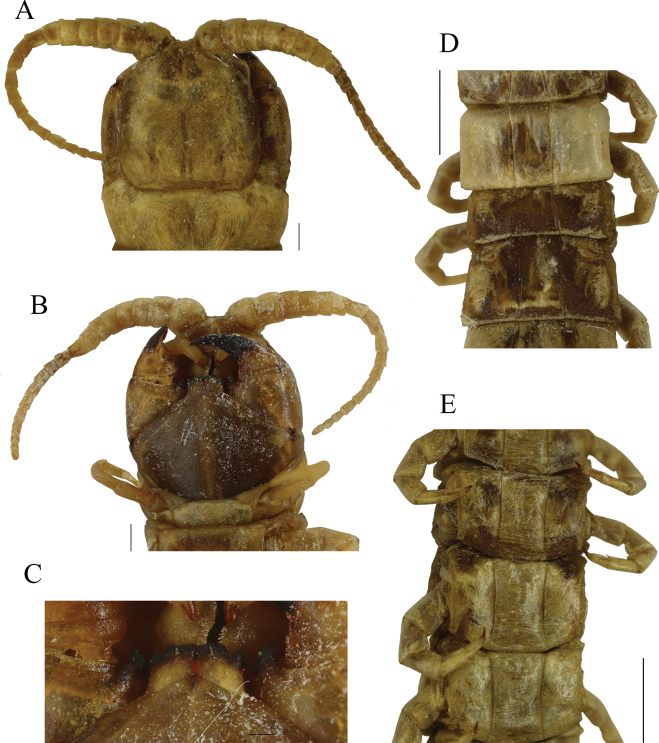
*Scolopendraoraniensis* syntype “1"; unsexed; (MNHN) **A** cephalic plate, antennae, and T1 **B** forcipular segment and sternites 1, 2 **C** tooth plates **D** tergites 3–6 **E** sternites 6–8. Scale bars: 0.2 mm (**C**); 1 mm (**A, B**); 2 mm (**D, E**).

**Figure 8.**
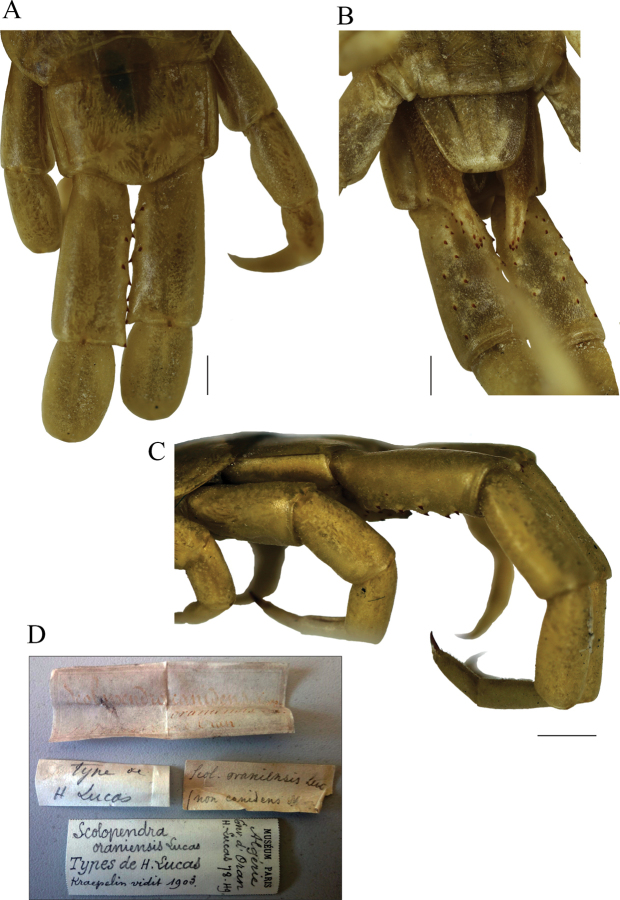
*Scolopendraoraniensis* syntype “1"; unsexed; (MNHN) **A** dorsal view of ULBS and UL**B** ventral view of ULBS and UL**C** lateral view of ULBS and UL**D** Original manuscript labels ordered chronologically. Top: old label [*Scolopendracanidens* Newp. *oraniensis* Luc. H. Lucas. [Leg.] Oran [type locality]; Middle: Kraepelin’s 1903 labels [“Type de H. Lucas. *Scol.oraniensis* Luc. (non *canidens* Newp.)”]. Lower: MNHN staff “modern” summary label. Scale bars: 1 mm (**A, B**); 2 mm (**C**).

**MNHN**: **Algeria** • 7 unsexed adults (2 probable females) *S.oraniensis* (type series); Oran’s wilayah; Santon’s mountains (corrected from “Sauton” (sic.) Lucas 1846 (ca 35°44'03"N, 7°08'16"W) and between Oran and Mers-el-Kebir (ca 35°43'40"N, 0°42'29"W); P.H. Lucas Leg. In ravines, lapidicolous (Figs 7, 8; Table 3).

## ﻿Systematics

**Order Scolopendromorpha Pocock, 1895**

**Family Scolopendridae Leach, 1814**

**Subfamily Scolopendrinae Kraepelin, 1903**

**Tribe Scolopendrini Leach, 1814**

**Genus *Scolopendra* Linnaeus, 1758**

### ﻿ Scolopendra viridipes

Taxon classificationAnimaliaScolopendromorphaScolopendridae

Dufour, 1820

E1E44B1A-5FD6-5335-939D-5DBF0578D0C4

**Figure 9.**
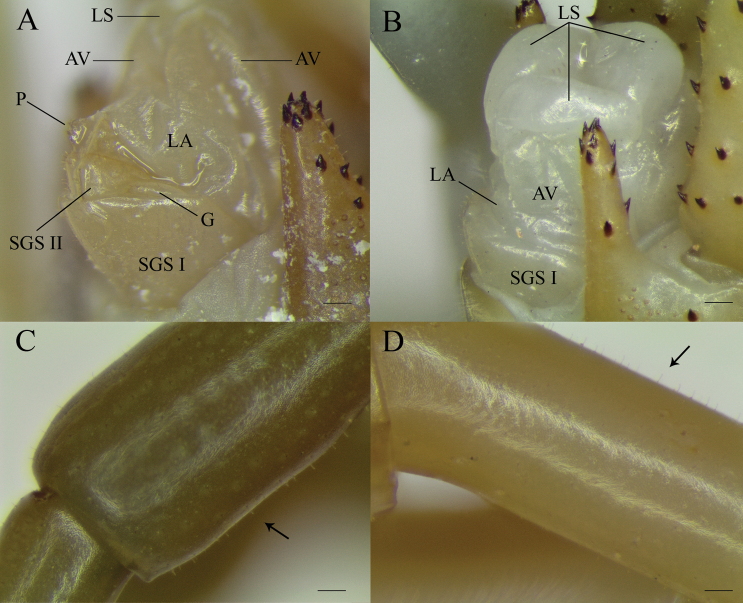
*Scolopendraoraniensis* genitalia **A** male (CEUAMr24); and **B** female (CEUAMr31), and detail of the setae (arrows) on tarsus 1 (**C, D)** on the same specimens **C** male and **D** female. Observe that on the contrary that Würmli (1980) indicated, the numbers of setae are actually similar in both genders. Legend: AV, anal valve; G. gonopod (folded back); LA, lamina adanalis; LS, lamina subanalis; P, penis; SGS I, sternite of genital segment 1; SGS II, sternite of genital segment 2. Scale bars: 0.1 mm.

Figs 2B , 3 , 4 , 9A, C , Table 3 , Suppl. material 1: file 1

Scolopendra viridipes Dufour, 1820: 317.
?S.clavipes C. L. Koch, 1836 [1847 sic.]: Brandt (1840): 149; Ranzani (1841): 441.
S. viridipes : Walckenaer and Gervais 1841: 258, as unrecognisable taxon.
?S.doriae Pirotta, 1878a (= S.cingulata): Pirotta 1878b: 406.
?S.oraniensisLucas 1846: Kraepelin 1903: 246, as “?S.viridipes Dufour, 1860" (sic.).
S. viridipes [Dufour, 1822 sic.]: Attems 1930: 51, as unrecognisable taxon.

#### Etymology.

From Latin *viridis* (green) and *pes* (feet), literally meaning green-footed *Scolopendra*.

#### Type series and type depository.

Types currently lost.

#### Collector and collection date.

J. M. L. Dufour, between 1811 and 1813 (Dufour 1888; Hessel 2000; Ferrández 2020).

#### Type locality.

“Kingdom of Valencia”, Valencian Community, east of Spain.

#### Distribution.

As for type locality.

#### Neotype designation.

With the express purpose of clarifying the taxonomic status and the type locality of the nominal taxon *S.viridipes* Dufour, 1820, the following neotype for this species is designated (ICZN 1999: Art. 75 and 76.3):

Male. Embassament del Bosquet, Moixent; Valencia Province (Spain) (38°51'21.7"N, 0°44'36.7"W 380 m a.s.l.) (Figs 3, 4; Table 3; Suppl. material 1: file 1). Coll. 26 Sep. 2020. C. Doménech leg. Repository in CEUA with the collection number CEUAMr21 (In this text also referred to as *S.viridipes* “topotype 1" before its neotype designation).

#### Proposed new nomenclatural status.

*S.viridipes* Dufour, 1820 is an invalid name subjectively designated here as nomen oblitum and a senior synonym of *S.oraniensis* Lucas, 1846.

#### Translation of the original descriptions from Latin and French.

[annotations in brackets]. (Original description available from: https://books.google.be/books?vid=GENT900000003803&printsec=frontcover#v=onepage&q&f=false)

XII. Green-footed Scolopendra

*Scolopendra* (viridipes) shell [dorsal habitus; tergites] livid, antennae and feet [legs] greenish, 21 feet [legs] in each side, the posterior ones longer.

Habitat: under the rocks of the Valencian Kingdom’s mountains. Length 18 lignes [1 Paris ligne = 2.2556 mm; 40.6 mm].

It differs from *Scol.morsitans*. The body segments [sensu tergites] are roughly square and equal between them, except for the first two and the last one. The head [cephalic plate] is small and oval. The whole body [dorsal habitus] has a markedly livid colour. The legs and the antennae are greenish. They [antennae] end in a setaceous point and have more than fifteen articles. The legs grow from the head [cephalic plate] to the anus [ultimate leg-bearing segment; ULBS]. The palps [first maxillary telopodites] end with a dilated and round article [article 3].

#### Remarks involving the type series and type series depository.

Efforts to locate *S.viridipes* type series in its four most probable repositories, CLD, MNHN, MNCN, and SLB, were unsuccessful (J. J. Geoffroy (MNHN) and B. Sánchez (MNCN) pers. comm. May 2020 to Oct 2021; CD pers. obs.).

Knowing that Dufour mostly preserved his specimens in dry conditions (Dufour 1888; Ferrández 2020), it is conceivable that the major part of his material was degraded by subsequent insufficient curatorial handling (Ferrández 2020; MA Ferrández pers. comm. May 2020–Oct. 2021).

Therefore, the *S.viridipes* type series is here considered as definitively lost material.

#### Original description comparison.

Because the type material for *S.viridipes* has vanished, and to determine to which species Dufour (1820) was referring, the available characters for this taxon are compared with all other Scolopendromorpha from Peninsular Spain (Table 1).

Initially, the brief morphological description does not allow one to assign *S.viridipes* to any definite genus because some features are common for all Scolopendromorpha, either because they are insufficiently detailed or widely observed (See “*” in Table 1). Cryptops (C.) lobatus Verhoeff, 1931, C. (T.) longicornis (Ribaut, 1915), as well as *Plutoniumzwierleini* Cavanna, 1881 should be discarded as candidates for the application of the name *S.viridipes*, due to the fact that their restricted distributional areas are outside of those of this species.

The six remaining *Cryptops* species (Fig. 1B) are smaller than *S.viridipes*, except for C. (C.) anomalans Newport, 1844. This rules out five of those taxa as candidates for this name (Table 1). *Theatopserythrocephalus* (C. L. Koch, 1847) can be similar in size to *S.viridipes*; however, it can be assumed that Dufour was actually not referring to *T.erythrocephalus* when he described *S.viridipes*, since he did not highlighted the conspicuous ultimate leg (UL) width, but pointed out a longer length; nor did he highlight the enlarged size of segment 21, remarking instead on the different sizes and shapes of TT1, 2, and 21, being these presumably smaller than the TT3–20 (Table 1). These are all distinctive traits for *T.erythrocephalus* (Fig. 1A).

Otherwise the exclusive colouration of *S.viridipes*, a “livid” dorsal habitus with greenish legs and antennae (Figs 2B, 3, 4), is an exclusive feature that clearly distinguishes it from all other Iberian Scolopendromorpha, with one exception, *S.oraniensis*. This colouration eliminates *T.erythrocephalus* and all the Iberian cryptopids as candidates because they all have consistently pale yellowish to reddish integuments and appendages, with no greenish pigmentation (Fig. 1A, B).

*Scolopendracingulata* always has tergites with posterior transverse black pigmentation combined with shiny red, orange, or yellow colouration in their legs when juvenile (Voigtländer and Reip 2013), or pale yellow legs when in an adult stage (Fig. 1C). Rarely, some adult individuals of *S.cingulata* from central and western Spain can exhibit a barely discernible green shade on the anterior and/or posterior locomotory legs and basal antennae articles (i.e., Cabanillas and García-Febrero 2020). This pigmentation is absent in the populations surrounding València, the type locality of *S.viridipes* (Fig. 2A; BV 2024).

The remaining Scolopendromorpha species inhabiting the presumed type locality of *S.viridipes* is *S.oraniensis*. This species is variable in colouration even within local populations (Fig. 2D–I; BV 2024); however, the pale and greenish habitus is by far one of the most frequent variations in Valencia. Therefore, because of its compatible distribution, size, morphology, and colouration, *S.oraniensis* is a good candidate to be the species to which Dufour was actually referring under the name *S.viridipes* (Table 1, Suppl. material 1: file 1).

Hence, prior to analysing some compatible topotypic material, all features stated in the original description strongly suggest that the closest relative to *S.viridipes* is *S.oraniensis*, if they do not belong to the same taxon.

#### Neotype and “topotypes” morphological comparisons.

Four colour-, morphological-, and size-compatible *S.viridipes* topotypes were collected in two Valencian localities known by their authorities (Figs 2B, 3, 4). All these specimens were examined and confirmed to be conspecific (Suppl. material 1: file 1). From those a neotype was selected (ICZN 1999: Art. 75; Figs 3, 4; Table 3; Suppl. material 1: file 1). The comparison of the neotype and other “topotypes” with the syntypes of the closest relative, *S.oraniensis* (Table 1) confirmed that all of them shared identical diagnostic features (Table 3; compare Figs 3, 4 with Figs 7, 8). Hence, according to all the data presented above, *S.viridipes* and *S.oraniensis* are here designated to be conspecific taxa.

#### Taxonomic and nomenclatural status.

As the two taxa, *S.viridipes* Dufour, 1820 and *S.oraniensis* Lucas, 1846, are deemed to be conspecific, the “Principle of Priority” provides preference for the name *S.viridipes* to replace the name *S.oraniensis* (ICZN 1999: Art. 23, 23.2, 23.3). However, we propose the nomenclatural reversal of precedence (ICZN 1999: Art. 23) in favour of the prevailing usage and nomenclatural stability of the largely accepted name *S.oraniensis* since the two conditions in Art. 23.9 (ICZN 1999) are met:

1. “the senior synonym [*S.viridipes*] […] has not been used as a valid name after 1899" (ICZN 1999: Art. 23.9.1.1).

This statement is not strictly true, since Kraepelin (1903) and Attems (1930) did use that name after 1899. Nevertheless, the use of the name in those two publications satisfy the Art. 23.9.6 (ICZN 1999), which clarifies that “the mentioning of a name in a synonymy [Kraepelin 1903], or [...] list of names [Attems 1930] must not be taken into account in determining usage under Art. 23.9.1.1 and 23.9.1.2".

1. “ [*S.oraniensis*] has been used [...] as its presumed valid name, in at least 25 works, published by at least ten authors in the immediately preceding 50 years and encompassing a span of not less than ten years” (ICZN 1999: Art. 23.9.1.2; see Suppl. material 1: file 2).

Hence, the name *S.viridipes* is here declared invalid since this is subjectively considered a senior synonym and nomen oblitum of *S.oraniensis*, while the name *S.oraniensis* is proposed as a nomen protectum, being fixed for unequivocal referencing of this species (ICZN 1999: Art. 23.9.2).

### ﻿ Scolopendra chlorotes

Taxon classificationAnimaliaScolopendromorphaScolopendridae

L. Koch in Rosenhauer, 1856

EA156D06-F21B-585F-9BDF-75B11DEB39C1

Figs 2D, E , 5 , 6 , 9B, D , Table 3 , Suppl. material 1: file 3

Scolopendra chlorotes L. Koch in Rosenhauer, 1856: 417.
?S.oraniensis Lucas, 1846: Kraepelin 1903: 246.
S. chlorotes : Attems 1930: 49, as unrecognisable taxon.

#### Etymology.

From the Greek word *χλοερός* (*khloerós*, verdant) and *χλόη* (*khlóē*, “the green of new growth”) meaning greenish yellow, pale green, pale, pallid, or verdant, referencing their pale greenish and yellowish leg colouration.

#### Type series and type depository.

Types currently lost.

#### Collector and collection date.

W. G. Rosenhauer, in 1849 (Rosenhauer 1856).

#### Type locality.

“Near Málaga”, Andalusia, Spain.

#### Distribution.

As for type locality.

#### Neotype designation.

With the express purpose of clarifying the taxonomic status and the type locality of the nominal taxon *S.chlorotes* L. Koch in Rosenhauer, 1856, the following neotype is designated (ICZN 1999: Art. 75 and 76.3):

Male. Carretera de Coín, Alahurín de la Torre, Málaga province (Spain) (36°39'37'’N, 4°31'0.4'’W 104 m a.s.l.) (Figs 5, 6; Table 3; Suppl. material 1: file 1). Coll. 18 Nov. 2021. C. Doménech Leg. Repository CEUA with the collection number CEUAMr29 (In this text also referred as “*S.chlorotes* topotype 5" before its neotype designation).

#### Proposed new nomenclatural status.

*Scolopendrachlorotes* L. Koch in Rosenhauer, 1856 is an invalid name, here subjectively proposed as a junior synonym of *Scolopendraoraniensis* Lucas, 1846.

#### Translation of the original descriptions from German

[annotations in brackets]. (Original description available from: https://www.biodiversitylibrary.org/page/42185817#page/425/mode/1up)

*Scolopendrachlorotes* Koch.

Brownish green, feeding pliers [forcipules], end-shield [ULBS tergite] and last pair of legs [terminal legs] reddish brown, on the last seven tergites, a furrow at the lateral edges [margination in tergites], on the first podomere of the last pair of legs [UL prefemur] 19 small teeth on the underside [ventral position], seven small teeth directed inward on the upper-side [medial and dorsomedial positions].

Length 20 ‘lignes’ [1 German – Nuremberg – lignes ≈ 2.11 mm; 42.22 mm].

Shiny; head [cephalic plate] longish, rather narrow, the head area dentate [probably referring to tooth plates] in the middle, tergite sides straight, those of the 3^rd^ and the penultimate tergite rounded anteriorly, the seven last tergites with a furrow at the lateral edges [tergite margination]. Tergites with the two normal stripes [paramedian sutures], except the first and the last tergites; the end-shield [ULBS tergite] shows a distinct median longitudinal furrow [suture]. The sternites have two longitudinal furrows [paramedian sutures], except the last sternite. The last pair of legs short, dorsally flat, the tooth-like process at the inner angle of the first podomere short [UL prefemoral process] with two blunt teeth [spines] at the tip; at the inner side [medial position] of this podomere [UL prefemur] seven small teeth [spines] at the upper side [medial position], 19 small teeth [spines] arranged in four rows at the underside [ventral position]. Head [cephalic plate], antennae, and tergites, except the last one, brownish green, the first podomeres [prefemur, femur, and even tibia] of the legs yellowish, the last [tibia and tarsi 1 and 2] green; maxillipeds, lower lip [probably trochanterprefemoral parts of the forcipula, tooth plates or/and coxosternite], end-shield [ULBS tergite] and last pair of legs [UL] reddish brown, the capture-claw [forcipules] brownish black from the middle [tarsungula].

Near Malaga, sporadic.

#### Remarks involving the type series and type series depository.

All attempts to locate the type series of *S.chlorotes* were unsuccessful; the curators in their four probable depositories (NHMN, NHMUK, ZMB, and FAU) found no samples in their collections (pers. comm. Aug. 2020 to Nov. 2021).

According to Rühm (1925) and Hessel (2000) the types of this species most probable depository should be the NHMUK, on the basis that in 1925 the Naturhistorische Gesellschaft (NHG), lacking financial resources, decided to move a large number of their specimens preserved in ethanol to the British Museum of Natural History. Nevertheless, the types of *S.chlorotes* are not registered nor found there.

The other smaller part of Koch’s wet collection, probably containing this species type series, did remain at the NHMN. However, the NHG rooms, their catalogues, and a large part of the collections were damaged during World War II in 1945 and presumably also the *S.chlorotes* types (EM Neupert (NHG) pers. comm. Sep. 2020). Therefore, the type series of *S.chlorotes* is considered to be lost.

#### Original description comparison.

Because the type material is unavailable, the features in the original description are compared to those of other Scolopendromorpha found in Peninsular Spain to determine to which taxon L. Koch was referring when he erected *S.chlorotes* as a new species (Table 2). In this case, the original description of *S.chlorotes* allows exclusion of all *Cryptops* species because the sternite sutures are cruciform or trigonal (rather than only the two paramedial ones) and the UL prefemoral spines and (most likely referred to as) coxosternal tooth plates are absent in this genus (Schileyko et al. 2020). Neither of the two Mediterranean representatives of the family Plutoniumidae match with the morphological description of *S.chlorotes* since both have sternites with a distinctive single medial longitudinal suture and none, or just one, spine on UL prefemur (Bonato et al. 2017b). The compatibility of *S.cingulata* is also rejected due to the absence of the longitudinal suture on the T21 with the incompatibilities in the prefemoral spinulation of the UL (Table 2). Furthermore, all of these taxa can be also ruled out because their colours do not match with those of *S.chlorotes* (Table 2).

Hence, its unambiguous morphology and colouration, compatible location, and the exclusion of all other Iberian Scolopendromorpha are facts that, combined, strongly suggest that if it is not the same taxon, the closest relative to *S.chlorotes* is *S.oraniensis* (Table 2).

#### Neotype and “topotypes” morphological comparisons.

A total of fifteen *S.chlorotes* topotypical specimens were collected in five municipalities “near Málaga” (L. Koch 1856; Figs 2A, D, E, 5, 6; Suppl. material 1: file 3). All these specimens were examined and confirmed to be conspecific (Suppl. material 1: file 3). Among those, a neotype was selected (ICZN 1999: Art. 75; Figs 5, 6, Table 3, Suppl. material 1: file 3). The comparison of the neotype and the other “topotypes” with the seven syntypes of the closest taxon *S.oraniensis* demonstrated that all these specimens possess identical diagnostic morphological features (see diagnosis and redescription above; Table 3; compare Figs 5, 6 with Figs 7, 8) and therefore, both taxa should be considered to be the same species.

Additionally, the neotype of *S.chlorotes* and the other “topotypes” were compared with those of *S.viridipes*, and conspecificity was also confirmed (compare Figs 3, 4 with Figs 5, 6; Table 3). Consequently, *S.chlorotes*, *S.viridipes*, and *S.oraniensis* are considered a single taxon with three names (Figs 3–8; Table 3).

#### Taxonomic and nomenclatural status.

As long as the taxon *S.chlorotes* L. Koch in Rosenhauer, 1856 is recognised as conspecific with *S.oraniensis* Lucas, 1846, the principle of priority indicates that the valid name of *S.chlorotes* is *S.oraniensis* (ICZN 1999: Art. 23.3). Hence, *S.chlorotes* is subjectively designated as an invalid name since it is here considered a junior synonym of *S.oraniensis* (ICZN 1999: Art. 23.3).

### ﻿ Scolopendra oraniensis

Taxon classificationAnimaliaScolopendromorphaScolopendridae

Lucas, 1846

C03A6625-891E-551F-B071-36160948D577

Types: Figs 7 , 8 ; Table 3 . Non-types: Figs 1C–I , 2B, D , 3 , 4 , 5 , 6 , 7 , 8 , 9 ; Tables 1 , 2 , 3 ; Suppl. material 1: files 1, 3

Scolopendra viridipes Dufour, 1820; nomen oblitum; senior syn. nov.
S. oraniensis Lucas, 1846; nomen protectum.
S. chlorotes L. Koch in Rosenhauer, 1856; junior syn. nov.
S. mediterranea Verhoeff, 1893: 318.
S. mediterranea lusitanica Verhoeff, 1893: 318.
S. clavipes Silvestri, 1897: 7 (sic.).
S. oraniensis africana Attems, 1902: 556.
Rhadinoscytalis canidens oraniensis : Attems 1926: 246.
S. canidens oraniensis : Attems 1930: 19, 36, 37, fig. 50.
S. canidens lusitanica Verhoeff, 1931: 309.
S. oraniensis : Würmli 1980: 348–350.

#### Morphological diagnosis

[**based on *S.oraniensis* type series].** Body length up to 64 mm. 18 or 19 antennal articles; 5½ basal ones glabrous in their entire surface. Antennae/cephalic plate length ratio ≈ 3.30. Forcipular trochanteroprefemoral process clearly defined, with two or three rather inconspicuous denticles. Tooth plate with 4+4 (rarely 3+3) teeth, divided into two groups. T1 without sutures or sulci. Paramedian sutures complete on tergites TT2–20. T21 with a complete median longitudinal suture. Margination starting at T14, complete on TT19–21. Sternite paramedian sutures on TT2–20. Coxopleuron basally pore-field, with a single medio-distal spine. Coxopleural process with a small pore-field, sub-cylindrical and quite elongated, with 9–14 spines altogether, disposed in sub/apical, dorsal, or lateral positions. Legs 3–20 with few setae. Two tarsal spurs on legs 1; a single tarsal spur on legs 2–18 or 19. Ultimate leg elongated, sometimes with a sinusoid transverse sulcus on the ventro-distal part; prefemoral spines (usually between 18–28) arranged in five frequently miss-aligned or duplicated rows with the VL: 1-5, V: 2-6, VM: 2-6, M: 4-8 and DM: 2-4 formula. Prefemoral process inconspicuous ending with two or three (rarely four) spines. Prefemur and femur of UL glabrous; tibia distally covered by sparse setae; tarsi 1 and 2 covered by small setae. UL/T21 length ratio ≈ 5.15.

#### Etymology.

from the toponym “Oran”, Algeria and the feminine (or masculine) suffix -*ensis* (from) meaning “from Oran”, Algeria, in reference to the type locality.

#### Type series and type depository.

Lucas (1846) did not declare on which specimens he based the nominal taxon of *S.oraniensis* (type series) (ICZN 1999: Art. 72.4.1), neither exposing the specimens’ depository nor designating a holotype (ICZN 1999: Art. 72.1.1). Otherwise, Kraepelin (1904) indicated the presence of the types [syntypes] at the MNHN in writing “– [*Scolopendra*] *oraniensis* Lucas. – Algérie: envir. d’Oran [surroundings of Oran] (H. Lucas, 1849 [sic.]. Types)”, without further data. Therefore, after the examination of the MNHN specimens and their labels (Figs 7, 8) (ICZN 1999: Art. 72.4.2), I conclude that the type series for the *S.oraniensis* nominal taxon was erected on the basis of seven syntypes, all of them unsexed adults (Table 3). Depository MNHN, Paris, France. Jar Number 282. Samples (Figs 7, 8A–C) and labels (Fig. 8D) separated in three different assay tubes (containing 2/2/3 specimens, respectively).

#### Collector and collection date.

P. H. Lucas, during winter, between 1839 and 1842 (Lucas 1846).

#### Type locality and habitat

from Lucas 1846: Ravines of the “Sauton’s” (sic.) [Santon] mountains and ravines between Oran and Mers-el-Kebir, Oran wilayah; Algeria (ICZN 1999: Recommendation 76A.2). Lapidicolous.

#### Distribution.

Known from southern France (including Corsica), southern Italy (including Sardinia and Sicilia), Malta, Spain (including Balearic Islands), Portugal, Morocco, and Algeria. Introduced in Japan (Attems 1930; Würmli 1980; Bonato et al. 2017a).

#### Proposed new nomenclatural status.

Nomen protectum.

#### Type series composite redescription

**(Table 3). *Colouration***: Colouration of specimens preserved in ethanol is toasted to pale yellow (Figs 7, 8A–C). According to Lucas (1846), colouration of living specimen was as follows [morphological traits are interpreted in brackets]:

(Original colour description available from: https://www.biodiversitylibrary.org/page/2362627#page/301/mode/1up)

Upper body part [anterior tergites] coppery black, lower [posterior tergites] green, in the middle ornamented with a yellowish green longitudinal stripe [probably, the translucence of the Malpighian tubule], [...] jaw [forcipules] reddish [...] palps greenish [second maxillae]; base of antenna green, in the middle greenish and in front stained dark red; feet [locomotory legs] green with dark red nails [unguis proper]; last pair of legs [UL] dark green [...].

#### Morphological description of the type series.

[notes on brackets are comprehensive annotations from this text author]: Body length up to 64 mm. Antennae reaching up to T2 [maybe up to T3; actual length shrivelled by ethanol retraction]; with 18 or 19 articles, the basal 5½ ones dorsally and ventrally glabrous (Fig. 7A, B). Antennae/cephalic plate length ratio: 2.01 [reduced because of the antennae articles retraction caused by the preservation in ethanol; estimated to be ~ 3.30].

Cephalic plate with disperse puncta and a short anterior longitudinal depression; disto-median or/and paramedian sutures are absent. Posterior edge of cephalic plate overlapping the T1 (Fig. 7A). Coxosternal surface with disperse puncta, without sutures (Fig. 7B). Article 2 of second maxillary telopodite with spur. Forcipular trochanteroprefemoral process with one apical and two (rarely one) medial poorly differentiable teeth (Fig. 7C). Tooth plates slightly wider than tall, forming an obtuse angle (> 120°) with respect to the coxosternite; sensillae present. Each tooth plate with 4+4, rarely 3+3 teeth: the external one separated from the other three and inner ones sometimes fused. (Fig. 7C).

Spiracles triangular with three valves, present on body segments 3, 5, 8, 10, 12, 14, 16, 18, and 20.

T1 without sutures or sulcus (Fig. 7A); TT2–20 with complete paramedian sutures (Fig. 7D); T21 with a longitudinal median suture, surface of all tergites smooth, without depressions (Fig. 8A). T21 width/length ratio ≈ 1.35. Margination starting at T14, being complete in TT19–21.

Sternites with complete paramedian sutures from TT2–20 (Fig. 7E). Sternite of ultimate leg-bearing segment (Fig. 8B) with sides converging posteriorly; surface smooth, without depressions.

Coxopleuron not surpassing the posterior border of the tergite of the ULBS (Fig. 8B); with a dense pore-field area at the base and a single medio-distal spine. Coxopleural process sub-cylindrical and distinctly elongated, reaching up to the first 1/4 of the UL prefemoral length. The complete surface is covered by a loose pore-field with some small setae (Fig. 8C) and a total of 9–14 usually asymmetrically disposed spines; 4–6 of them in an apical/subapical position; 3–6 in a lateral position, and 1–3 in a dorsal position.

Leg 1 with two distal tarsal spurs on tarsus 1, one lateral anterior and one ventral; legs 1–18 or 19 (mode 19, Table 3) with only one ventral tarsal spur. Tibial spurs absent. Legs 1, 2, or 3 with or without isolated setae. Legs 3 or 4–20 with scarce setae.

UL moderately long and slender with ratios of lengths of prefemur and femur = 1.15, femur and tibia = 1.25, tibia and tarsus 2 = 1.80; tarsus 1 and tarsus 2 = 1.20 (Fig. 8C). Prefemora flattened dorsally, sometimes with a visible sinusoid transverse sulcus on the ventro-distal side (Fig. 8A, B). Spines are variable in number (total: 17–26) and size, usually arranged in five frequently asymmetrical, duplicate, and non-ordered rows, with a formula: VL: 3-6, V: 2-6, VM: 2-6, M: 3-8 and DM: 2-4. VM and M spine rows usually converging proximally; between them a flattened surface is present, regularly rounded proximally and without spines. Prefemoral process inconspicuously prominent, sometimes scarcely setose, ending with two, or rarely three, spines (Figs 8A–C). Setae on prefemur and femur almost absent, tibia distally scarce setose and tarsi 1 and 2 densely covered with short setae. UL/T21 length ratio ≈ 4.39 [reduced because of the retraction of the articles caused by the preservation in ethanol; ~ 5.15].

Genitalia retracted in the whole type series.

#### Genitalia description.

(Based on *S.viridipes* and *S.chlorotes* “topotypical” material; Fig. 9A–D).

Genitalia well developed, reaching the apical part of coxopleural process when extended. Sternite and tergite of genital segment 1 (TGS and SGS I) convex and distally round, with a proximal median suture – distally attenuated – forming the vertex of a poorly angulated keel (Fig. 9A, B). In males (Fig. 9A) tergite of genital segment 2 (SGS II) small and horseshoe-shaped, with a poorly visible longitudinal suture. Gonopods short and small. Penis present, with fee endpoint, ventally serrated. TGS, SGS I, SGS II, lamina adanalis, gonopods and penis carrying scatted small setae, the remaining part of the genital structures glabrous. Sexual dimorphism or secondary sexual characteristics indistinct (Figs 9C, D); UL tibia and tarsi weakly and inconstantly more hirsute in females, with gender differences frequently indistinguishable.

#### Species variability.

*Scolopendraoraniensis* has been observed to be somewhat variable in some few morphological features (Figs 3–9B; Table 3, Suppl. material 1: files 1, 3): number of antennal articles (17–20; mode 18–19), number of hirsute basal antennal articles (5–5½), number of teeth on tooth plates and on forcipular trochanteroprefemoral processes (respectively 3+3 vs 4+4; mode 4+4 and 3 vs 2; mode 3), beginning of complete margination of tergites (on TT17, 19, or 20; mode 17), tarsal spurs on leg 1 (0-2; mode 2), single tarsal spurs on legs (2–18 or 19; mode 2–19), coxopleural process morphology and UL prefemur number of spines (respectively 9–14 and 18–32), visibility of UL prefemoral ventro-distal horizontal suture, coxopleural and prefemoral process lengths or number of setae on the UL tarsi.

Additionally, the colouration has proven to be variable sympatrically (Figs 1D–I, 3–9). The cephalic plate and tergites can be monochromatic pale yellow to dark green/brown, sometimes reddish, sometimes with a darker/lighter longitudinal stripe. The forcipules can be yellowish to reddish, while the legs and antennae can be pale green, yellowish, bluish, reddish, or bicoloured, with occasional metallic reflections (Figs 1D–I, 3, 9).

These morphological and colouration characteristics, although tending towards a certain geographic distribution, often turn out to be variable within those populations (Table 3, Suppl. material 1: files 1, 3). Therefore, the separation of the species by their old names are clearly unjustified, since all the features can be explained by the known intraspecific variability of the taxon (Iorio and Geoffroy 2006; García-Ruiz 2018), which is also shared with many other species in the genus (Zalesskaja and Schileyko 1992; Siriwut et al. 2016; Doménech et al. 2018; Dyachkov and Nedoev 2021; Tsukamoto et al. 2021).

#### Differential diagnosis.

Morphologically, *S.oraniensis* is related to five other species of the *S.canidens* group distributed around the Mediterranean Sea and Middle Asia (Würmli 1980; Simaiakis and Mylonas 2008). *Scolopendraclavipes* (Middle Asia) can easily be distinguished from *S.oraniensis* by the clavate morphology of the UL, while the two subspecies of *S.dalmatica* C. L. Koch, 1847 (northeastern Mediterranean) can be differentiated by their larger size (up to 80 mm), the incomplete median suture of T21, and the four spines of the UL prefemoral process. However, the morphologically closest relatives to *S.oraniensis* are *S.canidens* (southeastern and eastern Mediterranean) and *S.cretica* Lucas, 1853 (eastern Mediterranean, probably endemic to Crete, Greece). These two latter species can be unequivocally differentiated from *S.oraniensis* by the number of glabrous articles in the antennae (10–12 vs 5–5½, respectively), the type of transition between the glabrous/hirsute articles (gradual vs abrupt), and the number of spines in the prefemoral process (generally 3 vs 2 or 3). Between *S.canidens* and *S.cretica*, the only visible difference is the presence of brush-like setae in tarsi 1 and 2 of the UL in the *S.cretica* females (Würmli 1980; Lewis 2010; Dyachkov (ASU) pers. comm. 2022; Huesca pers. comm. 2018).

In these last two species closest to *S.oraniensis*, comparative analyses such as antennae/cephalic plates and T21/UL length ratios and their genitalia descriptions have not been performed yet.

#### Nomenclatural considerations.

To verify the actual identities of *S.viridipes* and *S.chlorotes*, establishing the *S.oraniensis* specimens Lucas (1846) on which he based the type series was necessary, to have reference material with which to unambiguously compare new material (ICZN 1999: Preamble, Art. 13, Recomm. 13A). The *S.oraniensis* lectotype designation was found to be not nomenclaturally required (ICZN 1999: 11; point 6 in introduction; Art. 72.1.1; 74.7). However, neotypes designations for *S.viridipes* and *S.chlorotes* were mandatory to clarify their type localities and taxonomic situations in the absence of the original types (ICZN 1999: Art. 75, 76). Finally, the identities of *S.viridipes*, *S.chlorotes*, and *S.oraniensis* have been solved, and they are all conspecific (Figs 3–8; Table 3) leaving the name *S.oraniensis* Lucas, 1846 as nomen protectum with which to unambiguously refer to “all” of these species (ICZN 1999: Art. 23.3 and 23.9.2).

At a nomenclatural level, the specific epithet of *S.oraniensis* obviously conformed to the reference of the type locality (Gervais 1847). However, that noun is composed by the feminine (or masculine) suffix “-*ensis*” (coming from) and the prefix (a toponym) “Orani”, in the place of “Oran”. Coincidentally, “Orani” is the name of two other localities located in Italy and the Philippines. This can lead to etymological confusion, especially for the Italian Orani, on Sardinia, a region where *S.oraniensis* also lives (Würmli 1980; Bonato et al. 2017a). Therefore, it is thought to be an incorrect original spelling (ICZN 1999: Art. 32.4) and according to the author’s intention to honour Oran, Algeria, rather than Orani, Italy, the proper name for *S.oraniensis* should be amended as “*S.oraniensis*”. However, ICZN (1999: Art. 32.5.1) clearly states that this supposition should not be adhered to since, in the original publication, “The […] use of an inappropriate connecting vowel is not to be considered inadvertent errors”. Therefore, despite being confusing or misspelled, this name must not be corrected as it is valid in its original spelling; *S.oraniensis*.

## ﻿Discussion

The identification of species that were described during the early time of binomial nomenclature (18^th^ and 19^th^ centuries) can often be a complicated task. The usually short and superficial descriptions as well as the frequent disappearance of type series are among the main reasons hindering these tasks. In that time, the limited access to information and collections, and the insufficient faunistic and taxonomic knowledge frequently caused the ignorance of some species names, or, on the contrary, the unintentional creation of many synonyms for the same taxon, with authors working individually in different countries with little or no communication. Since their description, the combination of these scenarios has affected the identities of *S.viridipes* and *S.chlorotes*.

After concluding on these species conspecificity with *S.oraniensis*, it can be observed that Lucas, when he described in 1846 his “greenish legged *Scolopendra*”, he accidentally overlooked the legs colour similarity between his “new” species and Dufour’s (1820) one, *S.viridipes*. Lucas certainly knew about the *S.viridipes* description (see Lucas 1840) when describing *S.oraniensis*, but either overlooked this taxon or maybe had a different interpretation of Dufour’s text. On the other hand, both Dufour (1820) and L. Koch (1856) named their respective species, *S.chlorotes* and *S.viridipes*, highlighting the greenish colouration of the legs. Despite this similarity in colour and size, morphology and relatively close distribution, L. Koch did not realise that he was facing a previously described species. In this context, the creation of this synonym could be justified since none of L. Koch’s previous literature supports the fact that this author was aware of the previous descriptions of *S.viridipes* or of *S.oraniensis*. Hence, the final result was that the same taxon was introduced under three different names.

Kraepelin (1903) accurately speculated about the identities of *S.viridipes*, *S.oraniensis*, and *S.chlorotes* when suggesting the probable synonymy of these three species. However, a remarkable transcription mistake in the *S.viridipes* description year “? *S.viridipes* Dufour, 1860 (sic.)” instead of 1820, probably caused this author to never question the priority of the name *S.oraniensis* as the actual senior name for this species (ICZN 1999: Art. 23). Since the reluctant treatment in Attems’ work (1930), no other study involving these nomina inquirenda *S.viridipes* and *S.chlorotes* has been performed until now.

So far, the incorporation of molecular data has provided interesting insights in to the systematics of *Scolopendra* (Oeyen et al. 2014; Siriwut et al. 2015, 2016; Doménech et al. 2018; Tsukamoto et al. 2021). In this context, and with a few methodologically questionable exceptions (Siriwut et al. 2016; Kang et al. 2017), *Scolopendra* has constantly demonstrated a good correlation between morphology and the often-used biomarkers, despite geographic barriers, distances, and the well-known intraspecific morphological variations (Zalesskaja and Schileyko 1992; Siriwut et al. 2015, 2016; Doménech et al. 2018; Han et al. 2018; Dyachkov and Nedoev 2021; Tsukamoto et al. 2021). On the other hand, for *S.oraniensis* only four sequence of three distinct genes belonging to two specimens from Italy and Morocco, are currently available (Vathera et al. 2013; Oeyen et al. 2014). However, to completely rule out the existence of cryptic speciation, the *S.viridipes* and *S.chlorotes* neotypes and related materials used in this text could be included in a future molecular work which might support the current “old school” taxonomic outcomes obtained here.

Even in the absence of modern methodologies and since the present contribution has proven useful, other authors are encouraged to continue producing studies with this classic taxonomic approach. These endeavours could help to improve the current Chilopoda knowledge by clarifying the identity of some taxa described long ago, as it has been the case of *S.viridipes*, *S.oraniensis*, and *S.chlorotes*.

## Supplementary Material

XML Treatment for Scolopendra viridipes

XML Treatment for Scolopendra chlorotes

XML Treatment for Scolopendra oraniensis

## ﻿Additional information

### Author ORCIDs

Carles Doménech https://orcid.org/0000-0003-1890-9434

### Data availability

All of the data that support the findings of this study are available in the main text or Supplementary Information.

## Supplementary materials

Supplementary material 1 Supplementary information Data type docx Explanation note **file 1.***Scolopendraviridipes* (=*S.oraniensis*) neotype and “topotypes” morphological comparisons. AP, apical spines. SAP, subapical spines. DS, dorsal spines. LS, lateral spines. VL, ventro-lateral spines. V, ventral spines. VM, ventro-median spines. M, median spines. DM, dorso-median spines. SP, prefemoral process spines. UL, ultimate legs. ULBS, ultimate leg-bearing segment. T, tergite. TT, tergites. S, sternite. SS, sternites. RG=Retracted genitalia. *= Not visible, damaged, or regenerated. **file 2.** List of references satisfying the second requisite of reversal precedence of the principle of priority (ICZN 1999: Art. 23.9.1.2), concerning the names S.viridipes Dufour, 1820 and S.oraniensis Lucas, 1846. **file 3.***Scolopendrachlorotes* neotype (= *S.oraniensis*) and “topotypes” morphological comparison. AP, apical spines. SAP, subapical spines. DS, dorsal spines. LS, lateral spines. VL, ventro-lateral spines. V, ventral spines. VM, ventro-median spines. M, median spines. DM, dorso-median spines. SP, prefemoral process spines. UL, ultimate legs. ULBS, ultimate leg-bearing segment. T, tergite. TT, tergites. S, sternite. SS, sternites. RG, Retracted genitalia. SN, Supernumerary spines between VM and M rows. *= Not visible, damaged, or regenerated. https://binary.pensoft.net/file/1101820This dataset is made available under the Open Database License (http://opendatacommons.org/licenses/odbl/1.0/). The Open Database License (ODbL) is a license agreement intended to allow users to freely share, modify, and use this Dataset while maintaining this same freedom for others, provided that the original source and author(s) are credited.Carles Doménech

## References

[B1] Attems C (1930) Myriopoda. 2. Scolopendromorpha. Das Tierreich. W. De Gruyter, Berlin and Leipzig 54, 308 pp. 10.1515/9783112373002

[B2] Bollman CH (1893) The Myriapoda of North America. Bulletin of the United States National Museum 46: 1–210. 10.5962/bhl.title.2787

[B3] Bonato L Minelli A (2014) ChilopodaGeophilomorpha of Europe: A revised list of species, with taxonomic and nomenclatorial notes. Zootaxa 3770(1): 1. 10.11646/zootaxa.3770.1.1 24871280

[B4] Bonato L Edgecombe GD Lewis JGE Minelli A Pereira L Shelley R Zapparoli M (2010) A common terminology for the external anatomy of centipedes (Chilopoda). ZooKeys 69: 17–51. 10.3897/zookeys.69.737 PMC3088443 21594038

[B5] Bonato L Chagas A Junior Edgecombe GD Lewis JGE Minelli A Pereira LA Shelley RM Stoev P Zapparoli M (2017a) ChiloBase 2.0. A World Catalogue of Centipedes (Chilopoda). http://chilobase.biologia.unipd.it [Accessed 10 Jan. 2020]

[B6] Bonato L Orlando M Zapparoli M Fusco G Bartolin F (2017b) New insights into *Plutonium*, one of the largest and least known European centipedes (Chilopoda): Distribution, evolution and morphology. Zoological Journal of the Linnean Society 180(4): 887–909. 10.1093/zoolinnean/zlw026

[B7] Brandt JF (1840) Observations sur les espèces qui composent le genre Scolopendra, suivies des charactères des espèces qui se trouvent dans le Muséum zoologique de l’ Académie des Sciences de St.-Petersbourg et de quelques coups d’ oeil sur leur distribution géographique. Bulletin Scientifique publié par l’Académie Impériale des Sciences de Saint-Pétersbourg 7(11): 147–160.

[B8] BV [Biodiversidad Virtual] (2024) Chilopoda. http://www.biodiversidadvirtual.org/ [accession: 21 Jan. 2024]

[B9] Cabanillas D (2019) Ampliación de la distribución de *Scolopendracingulata* Latreille, 1829 y *Scolopendraoraniensis* Lucas, 1846 (Chilopoda, Scolopendromorpha, Scolopendridae) en la Comunidad de Madrid (España). Boletin de la Asociacion Espanola de Entomologia 43: 55–77.

[B10] Cabanillas D García-Febrero O (2020) Primera cita de *Scolopendracingulata* Latreille, 1829 (Scolopendromorpha: Scolopendridae) en las Islas Baleares (España). Boletin de la Asociacion Espanola de Entomologia 44: 203–206.

[B11] Cabanillas D Parejo-Pulido D (2019) Primer registro de Lamyctes (Lamyctes) emarginatus (Newport, 1844) (Chilopoda: Lithobiomorpha: Henicopidae) en la Comunidad Autónoma de Extremadura y otras citas de la provincia de Badajoz (España). Boletin de la SEA 64: 307–311.

[B12] Cabanillas D Albatros A García-Ruiz A Rodríguez-Luque F (2019) First observation of filial cannibalism in *Scolopendracingulata* Latreille, 1829 (Chilopoda: Scolopendromorpha: Scolopendridae). Bulletin of the British Myriapod and Isopod Group 31: 26–33.

[B13] Carballo J Daza JL (1991) Contribución al estudio faunístico de la clase Chilopoda en Andalucía occidental. Arquivos do Museu Bocage. Nova Série 2(5): 79–116.

[B14] Cavanna F (1881) Nuovo genere (*Plutonium*) e nuova specie (*P.zwierleini*) di Scolopendridi. Bollettino della Società Entomologica Italiana 13: 169–178.

[B15] Demange JM Richard J (1969) Morphologie de l’appareil génital mâle des Scolopendromorphes et son importance en systèmatique (Myriapodes Chilopodes). Bulletin du Muséum National d’Histoire Naturelle 40: 968–983.

[B16] Di Z Cao Z Wu Y Yin S Edgecombe GE Li W (2010) Discovery of the centipede family Plutoniumidae (Chilopoda) in Asia: A new species of *Theatops* from China, and the taxonomic value of spiracle distributions in Scolopendromorpha. Zootaxa 2667(1): 51–63. 10.11646/zootaxa.2667.1.4

[B17] Doménech C Nagel P (2022) On the dates of publication of four European species of *Scolopendra* Linnaeus, 1758 described by C. L. Koch (Myriapoda, Chilopoda). Zootaxa 5159(1): 125–135. 10.11646/zootaxa.5159.1.6 36095555

[B18] Doménech C Barberá VM Larriba E (2018) A phylogenetic approach to the Philippines endemic centipedes of the genus *Scolopendra* Linnaeus, 1758 (Scolopendromorpha, Scolopendridae), with the description of a new species. Zootaxa 4483(3): 401–427. 10.11646/zootaxa.4483.3.1 30313775

[B19] Doménech C Cabanillas D Gilgado JD Garcia L Hernández-Corral J (2023) Contribución al conocimiento de los ciempiés, milpiés (Myriapoda: Chilopoda, Diplopoda) e isópodos terrestres (Crustacea: Oniscidea) de la Sierra de Aitana (Alicante, España). Arxius de Miscel·lània. Arxius de Miscel. Lania Zoologica 21: 129–149. 10.32800/amz.2023.21.0129

[B20] Dufour JML (1820) Description de dix espèces nouvelles ou peu connues d’Insectes recueillis en Espagne. Annales générales des sciences physiques 6: 307–317.

[B21] Dufour JML (1888) Souvenirs d’un savant français. A travers un siècle 1780–1865. J. Rothschild. Paris, 348 pp.

[B22] Dyachkov YV Nedoev KK (2021) A contribution to the centipede (Chilopoda: Geophilomorpha, Scolopendromorpha) fauna of Uzbekistan and Turkmenistan. Ecologica Montenegrina 41: 41–50. 10.37828/em.2021.41.6

[B23] Ferrández MA (2020) Léon Dufour y las arañas de España. Mundo Artropodo 8: 47–60.

[B24] García-Ruiz A (1993) Contribución al conocimiento de los quilópodos de la provincia de Jaén: I Puerto de Despeñaperros. Boletin del Grupo Entomologico de Madrid 6: 27–32.

[B25] García-Ruiz A (2007) Estudio biométrico de *Scolopendracingulata* Latreille, 1829 (Chilopoda, Scolopendromorpha). Boletin de la SEA 1(40): 542–544.

[B26] García-Ruiz A (2018) Contribución al conocimiento cavernícola de *Scolopendracanidens*, subsp. oraniensis (Lucas, 1846) (Myriapoda, Chilopoda). Monografías Bioespeleológicas 13: 13–16.

[B27] Gervais MP (1837) Etudes pour servir a l’histoire naturelle des Myriapodes. In Annales des Sciences Naturelles. série 2, Tome VII: 35–60. Ed. Crochard, Librairie-editeur, Paris.

[B28] Gervais MP (1847) Histoire naturelle des insectes. Aptères. Librairie encyclopédique de Roret, Paris, 623 pp.

[B29] Giribet G (2015) Orden Scolopendromorpha. Revista Ibero Diversidad Entomologica 30: 1–9. [Sociedad Española de Entomología]

[B30] Han T Lee YB Kim S-H Yoon H-J Park IG Park H (2018) Genetic variation of COI gene of the Korean medicinal centipede *Scolopendramutilans* Koch, 1878 (Scolopendromorpha: Scolopendridae). Entomological Research 48(6): 559–566. 10.1111/1748-5967.12331

[B31] Hessel B (2000) Dr. Karl Ludwig Christian Koch (8.11.1825 - 1.11.1908) Eine kurze Übersicht über das Leben und Wirken eines großen Nürnberger Naturforschers. Naturhistorische Gesellschaft Nürnberg e.V. 39–46.

[B32] ICZN (1999) International Code of Zoological Nomenclature. Fourth Edition. The International Trust for Zoological Nomenclature, London, UK. https://www.iczn.org/the-code/the-code-online/

[B33] Iorio É (2003) Morphologie externe des appareils génitaux mâle et femelle de la famille Scolopendridae (Chilopoda, Scolopendromorpha). Bulletin de Phyllie 16: 10–16.

[B34] Iorio É Geoffroy JJ (2006) Contribution à la connaissance de *Scolopendraoraniensis* H. Lucas, 1846 (Chilopoda, Scolopendromorpha, Scolopendridae). Le bulletin d’Arthropoda 27: 48–51.

[B35] Iorio É Geoffroy JJ (2008) Les scolopendromorphes de France (Chilopoda, Scolopendromorpha): identification et distribution géographique des espèces. Riviéra scientifique 91: 73–90.

[B36] Kang S Liu Y Zeng X Deng H Luo Y Chen K Chen S (2017) Taxonomy and identification of the genus *Scolopendra* in China using integrated methods of external morphology and molecular phylogenetics. Scientific Reports 7(1): 16032. 10.1038/s41598-017-15242-7 29167482 PMC5700134

[B37] Koch CL (1836) Deutschlands Crustaceen, Myriapoden und Arachniden. Ein Beitrag zur deutschen Fauna. Herausgegeben von Dr. Herrich-Schäffer. 9tes Heft. F. Pustet, Regensburg: Manz, 90 pp.

[B38] Koch CL (1847) System der Myriapoden. In: Herrich-Schäffer L (Ed.) Kritische Revision der Insectenfauna Deutschlands. Pustet, Regensburg, 270 pp.

[B39] Koch L (1856) . Myriapoda. In: Die Thiere Andalusiens. Rosenhauer WG (Ed.) Erlangen, 416 pp.

[B40] Kraepelin K (1903) Revision der Scolopendriden. Mitteilungen aus dem Naturhistorischen Museum in Hamburg 20(2): 1–276.

[B41] Kraepelin K (1904) Catalogue des scolopendrides des collections du muséum d’histroire naturelle de Paris. Bulletin du Muséum National d’Histoire Naturelle 26(10): 316–324.

[B42] Laboulbène M (1865) Liste de Travaux d’Entomologie Publiés de 1811 à 1864 Par M. Léon Dufour. Annales de la Société entomologique de France. Société entomologique de France 4(5): 216–252. 10.1080/00379271.1863.11755421

[B43] Lamarck (De) JBPA (1838) Histoire naturelle des animaux sans vertèbres: les caractères généraux et particuliers de ces animaux, leur distribution, leurs classes, leurs families, leurs genres, et la citation des principales espèces qui s’y rapportent; Tome cimquième. Arachnides, Crustacés, Annelides, Cirrihpedes. Deuxième Édition: revue et augmentée de notes présentant les faits nouveaux dont la science s’est enrichie jusqu’à ce jour; par mm. G. P. Deshayes et H. Milne Edwards. Ed. J. B. Baillière, Paris and London, 219 pp. 10.5962/bhl.title.23116

[B44] Lamarck (De) JBPA (1839) Histoire naturelle des animaux sans vertèbres: les caractères généraux et particuliers de ces animaux, leur distribution, leurs classes, leurs families, leurs genres, et la citation des principales espèces qui s’y rapportent; Tome deuxième. Arachnides, Crustacés, Annelides, Cirrihpedes. Troisième Édition: revue et augmentée de notes présentant les faits nouveaux dont la science s’est enrichie jusqu’à ce jour; par mm. G. P. Deshayes et H. Milne Edwards. Ed. Meline, Cans et Compagnie, Bruxeles, 698 pp. 10.5962/bhl.title.46287

[B45] Latreille M (1829) Les Myriapodes. Le régne animal distribué d’après son organisation, Nouvelle Édition, Revue et Augmentée - Tome IV - Crustacés, Arachnides et Partie des Insectes. Cuvier. PML, Paris 4: 326–339.

[B46] Leach WE (1814) Crustaceology. In: Brewster D (Ed.) The Edinburgh Encyclopaedia.Balfour, Edinburgh 7 (2), 383–437.

[B47] Lewis JGE (2010) A key and annotated list of the *Scolopendra* species of the Old World with a reappraisal of *Arthrorhabdus* (Chilopoda: Scolopendromorpha: Scolopendridae). International Journal of Myriapodology 3(1): 83–122. 10.1163/187525410X12578602960380

[B48] Lewis JGE (2011) A review of the species in the genus *Cryptops* Leach, 1815 from the Old World related to Cryptops (Cryptops) hortensis (Donovan, 1810) (Chilopoda, Scolopendromorpha). International Journal of Myriapodology 4: 11–50. 10.3897/ijm.4.1116

[B49] Linnaeus C (1758) Systema naturae. Editio Decima. Laurentius Salvius: Holmiae, 824 pp.

[B50] Linnaeus C (1767) Systema Naturae per regna tria nature, secundum classes, ordines, genera, species, cum characteribus, differentiis, synonymis, locis. Editio duodecima, reformata - Laurentius Salvius. Holmiae 2: 533–1327. 10.5962/bhl.title.158187

[B51] Lucas PH (1840) Histoire naturelle des crustacés, des arachnides et des myriapodes. P. Duménil, Paris, 600 pp. 10.5962/bhl.title.44485

[B52] Lucas PH (1846) Note sur quelques nouvelles espèces d’insectes qui habitent les possessions francaises du Nord de l’Afrique. Revue Zoologique 9: 283–289.

[B53] Lucas PH (1853) Essai sur les animaux articulés qui habitent l’ile de Crète - Revue et Magasin de Zoologie Pure et Appliquée 5(2): 514–531.

[B54] Machado A (1953) Alguns miriápodes de Espanha. Archivos Instituto de Aclimaticion 1: 77–92.

[B55] Matic Z Darabantu C (1968) Contributo alla conoscenza dei Chilopodi epimorfi (Chilopoda-Epimorpha) della fauna di Spagna. Memorie del Museo Civico di Storia Naturale di Verona 16: 127–135.

[B56] Minelli A (2011) Class Chilopoda, Class Symphyla and Class Pauropoda. In: Zhang, Z.-Q. (Ed.) Animal biodiversity: a higher level classification scheme and study of taxonomic richness. Zootaxa 3148(1): 157–158. 10.11646/zootaxa.3148.1.31

[B57] Moritz M Fischer SC (1979) Die Typen der Myriapoden-Sammlung des Zoologischen Museums Berlin. 11. Chilopoda. Mitteilungen aus dem Zoologischen Museum in Berlin 55(2): 297–352. 10.1002/mmnz.4830550215

[B58] NOAA/NWS (2022) National Oceanic and Atmospheric Administration, National Weather Service (NOAA/NWS 2022) website. www.noaa.gov [Accessed Nov. 2022; reproduced and modified according their licenses and given personal instructions; 7^th^ Dec. 2022]

[B59] Newport G (1844) A list of the species of Myriapoda order Chilopoda contained in the cabinets of the British Museum with synoptic descriptions of forty-seven new species. Annals & Magazine of Natural History 13(82): 94–101. 10.1080/03745484409442576

[B60] Oeyen JP Funke S Böhme W Wesener T (2014) The evolutionary history of the rediscovered Austrian population of the giant centipede *Scolopendracingulata* Latreille 1829 (Chilopoda, Scolopendromorpha). PLOS ONE 9(9): e108650. 10.1371/journal.pone.0108650 PMC4177219 25251436

[B61] Pirotta R (1878a) Miriapodi. Annali del Museo Civico di Storia Naturale di Genova XI: 1877–1878. [G. Doria e R. Gestro, Genova.]

[B62] Pirotta R (1878b) I miriapodi del “Violante”. Annali del Museo Civico di Storia Naturale di Genova 11: 397–410.

[B63] Ranzani C (1841) Rendiconto delle Sedunt dell’ Imp. Academie delle Scienze Di Pietroburgo. Nuovi annali delle scienze naturali 5: 394–445.

[B64] Ribaut H (1915) Biospeologica XXXVI. Notostigmophora, Scolopendromorpha, Geophilomorpha. Archives de Zoologie Expérimentale et Générale 55: 323–346.

[B65] Rosenhauer WG (1856) Die Thiere Andalusiens nach dem resulte einer Reise. Ed. Verlag von Theodor Blaesing, Erlangen, 429 pp. 10.5962/bhl.title.66016

[B66] Rühm J (1925) Der Neurenberger Naturforscher Dr. Ludwig Koch. Ein Gedenktblatt zum seinem 100. Geburstage am 8 Nov. 1925. Naturhistorischen Gesellschaft zu Nürnberg, 13 pp.

[B67] Schileyko AA Vathera V Edgecombe GD (2020) An overview of the extant genera and subgenera of the order Scolopendromorpha (Chilopoda): A new identification key and updated diagnoses. Zootaxa 4825(1): 1–64. 10.11646/zootaxa.4825.1.1 33056264

[B68] Serra A (1981) Contribución al conocimiento de Cryptops (Trigonocryptops) longicornis Ribaut (ChilopodaScolopendromorpha). Publicaciones del Departamento de Zoologia. Universidad de Barcelona 7: 47–50.

[B69] Serra A (1983) Els Scolopendrinae i els Theatopsinae (Chilopoda. Scolopendromorpha) de la Península Ibérica. Butlletí de la Institutició Catalana d’Historia Natural 49(5): 77–83.

[B70] Simaiakis S Mylonas M (2008) The *Scolopendra* species (Chilopoda: Scolopendromorpha: Scolopendridae) of Greece (E-Mediterranean): a theoretical approach on the effect of geography and palaeogeography on their distribution. Zootaxa 1792(1): 39–53. 10.11646/zootaxa.1792.1.3

[B71] Siriwut W Edgecombe GD Sutcharit C Panha S (2015) The centipede genus Scolopendra in mainland Southeast Asia: Molecular phylogenetics, geometric morphometrics and external morphology as tools for species delimitation. PLoS ONE 10(8): e0135355. 10.1371/journal.pone.0135355 PMC4536039 26270342

[B72] Siriwut W Edgecombe GD Sutcharit C Tongkerd P Panha S (2016) A taxonomic review of the centipede genus Scolopendra Linnaeus, 1758 (Scolopendromorpha, Scolopendridae) in mainland Southeast Asia, with description of a new species from Laos. ZooKeys 590: 1–124. 10.3897/zookeys.590.7950 PMC4926625 27408540

[B73] Tsukamoto S Hiruta SF Eguchi K Liao JR Shimano S (2021) A new amphibious species of the genus *Scolopendra* Linnaeus, 1758 (Scolopendromorpha, Scolopendridae) from the Ryukyu Archipelago and Taiwan. Zootaxa 4952(3): 465–494. 10.11646/zootaxa.4952.3.3 33903355

[B74] Vadell M (2013) Cryptops (Cryptops) lobatus Verhoeff, 1931 (Chilopoda: Scolopendromorpha: Cryptopidae), primera cita para la península ibérica. Boletin de la Asociacion Espanola de Entomologia 37: 1–2.

[B75] Vathera V Edgecombe GD Giribet G (2013) Phylogenetics of scolopendromorph centipedes: Can denser taxon sampling improve an artificial classification? Invertebrate Systematics 27(5): 578–602. 10.1071/IS13035

[B76] Verhoeff KW (1931) Über europäische *Cryptops*-Arten. Zoologische Jahrbucher. Abteilung fur Systematik, Ökologie und Geographie der Tiere 62: 263–288.

[B77] Verhoeff KW (1934) Beiträge zur Systematik und Geographie der Chilopoden. Zoologische Jahrbucher. Abteilung fur Systematik, Ökologie und Geographie der Tiere 66(1/2): 1–152.

[B78] Voigtländer K Reip HS (2013) Morphological, taxonomical and ecological contributions to the chilopod fauna of Andalusia (Sierra de Grazalema and Los Alcornocales), Spain. Graellsia 69(2): 217–241. 10.3989/graellsia.2013.v69.088

[B79] Walckenaer CA Gervais MP (1844) Histoire naturelle des insectes. Aptères. Tome Quatrième. Librairie encyclopédique de Roret, Paris, 623 pp. 10.5962/bhl.title.61095

[B80] Würmli M (1980) Statistische Unterschungen zur Systematik und postembryonalen Entwicklung der Scolopendracanidens Gruppe (Chilopoda: Scolopendromorpha). Sitzungsberichte der Österreichischen Akademie der Wissenschaften 189: 315–353.

[B81] Zalesskaja NT Schileyko AA (1992) The Distribution of Scolopendromorpha in the USSR (Chilopoda). Berichte des Naturwissenschaftlichen-Medizinischen Verein Innsbruck 10: 367–372.

