# Supplementary material for: ﻿Taxonomic and nomenclatural reassessment of the Iberian Peninsula’s nomina obscura, Scolopendraviridipes Dufour, 1820 and S.chlorotes L. Koch in Rosenhauer, 1856 (Chilopoda, Scolopendromorpha, Scolopendridae)
Source: Zookeys. 2024 Jul 25;1208:49–80. doi: 10.3897/zookeys.1208.122126 (PMC11294732; doi:10.3897/zookeys.1208.122126)
Supplement: Supplementary material 1 — Supplementary information [file zookeys-1208-049_article-122126__-s001.docx]

| **Supplementary File 1.** *Scolopendra viridipes* (=*S. oraniensis*) neotype and “topotype” morphological comparisons. AP, apical spines. SAP, subapical spines. DS, dorsal spines. LS, lateral spines. VL, ventro-lateral spines. V, ventral spines. VM, ventro-median spines. M, median spines. DM, dorso-median spines. SP, prefemoral process spines. UL, ultimate legs. ULBS, ultimate leg-bearing segment. T, tergite. TT, tergites. S, sternite. SS, sternites. RG=Retracted genitalia. *= Not visible, damaged, or regenerated. | | | | | |
| --- | --- | --- | --- | --- | --- |
|  | **Neotype**  **“Topotype 1” CEUAMr21** | **“Topotype 2”**  **CEUAMr22** | **“Topotype 3”**  **CEUAMr23** | **“Topotype 4” CEUAMr24** | ***S. viridipes***  ***“*Topotypes*”***  **(CEUAMr21-25)** |
| **Locality** | Moixent | Moixent | Xàtiva | Xàtiva | - |
| **Body length in mm** | 38 | 40 | 37 | 35 | 35-40 |
| **Sex** | Male | Female | Female | Male | - |
| **Compatible colouration with the original description** | Yes | Yes | Yes | Yes | Yes |
| **Antenna reaching to tergite** | T3 | T3 | T3 | T3 | T3 |
| **Number of antennal articles** | 19/19 | 20/14* | 17/18 | 18/18 | 17-20 |
| **Number of proximal glabrous articles** | 5 | 5 | 5 | 5 | 5 |
| **Teeth on tooth plate** | 4+4 | 4+4 | 4+4 | 4+4 | 4+4 |
| **Teeth on forcipular trochanteroprefemoral processes**  **Total (apical group/medial group)** | 3(2/1) - 3(2/1) | 3(2/1) - 3(2/1) | 3(2/1) - 3(2/1) | 3(2/1) - 3(2/1) | 3(1/2) |
| **Tergite paramedian sutures** | TT2-20 | TT2-20 | TT2-20 | TT2-20 | TT2-20 |
| **Longitudinal suture on T21** | Present | Present | Present | Present | Present |
| **First tergite with complete margination** | 17 | 20 | 17 | 17 | 17-19 |
| **Paramedian sutures on sternites** | SS2-20 | SS2-20 | SS2-20 | SS2-20 | SS2-20 |
| **Spines in coxopleuron**  **(Left/Right)** | 1/1 | 1/1 | 1/1 | 1/1 | 1/1 |
| **Spines in coxopleural process (Left/Right)** | AP-SAP: 4/5  DS: 1/2  LS: 3/2  Total:8/9 | AP-SAP: 6/4  DS: 1/2  LS: 2/2  Total:9/8 | AP-SAP: 6/6  DS: 2/2  LS: 3/2  Total:11/10 | AP-SAP: 8/6  DS: 3/1  LS: 3/2  Total:14/9 | AP-SAP: 4-8  DS: 1-3  LS: 2-3  Total: 7-14 |
| **Tarsal spurs on leg 1 (Left/Right)** | 2/2 | 2/2 | 2/0 | 1/1 | 0-2 |
| **Legs with one tarsal spur** | 2-19 | 2-19 | 2-19 | 2-19 | 2-19 |
| **Ultimate legs prefemoral spinulation and spines in prefemoral process (Left/Right)** | VL:6/6  V: 4/6  VM: 6/3  M: 6/8  DM:2/2  SP:3/2  Total: 27/27 | VL: 3/7  V: 6/6  VM: 3/3  M: 7/6  DM:2/2  SP:2/2  Total: 23/26 | VL:3/3  V: 5/6  VM: 4/6  M: 5/7  DM:2/2  SP:2/2  Total: 21/26 | VL:5/5  V: 5/4  VM: 7/6  M: 6/7  DM:2/2  SP:2/2  Total: 27/26 | VL: 3-7  V: 4-6  VM: 3-7  M: 5-8  DM: 2  SP: 2-3  Total: 19-33 |

**Supplementary File 2.** List of references satisfying the second requisite of reversal precedence of the principle of priority (ICZN 1999: Art. 23.9.1.2), concerning the names *S. viridipes* Dufour, 1820 and *S. oraniensis* Lucas, 1846.

1. Akkari N, Stoev P, Lewis J (2008) The scolopendromorph centipedes (Chilopoda, Scolopendromorpha) of Tunisia: taxonomy, distribution and habitats. ZooKeys 3: 77-102. doi: http://dx.doi.org/10.3897/zookeys.3.51
2. Cabanillas D (2021) High richness and unusual composition of centipedes in an urban renaturalised area of central Iberian Peninsula. Boletin de la Asociación Española de Entomología 45(3-4): 277-291.
3. Cabanillas D, García-Febrero O (2020) Primera cita de *Scolopendra cingulata* Latreille, 1829 (Scolopendromorpha: Scolopendridae) en las Islas Baleares (España). Boletin de la Asociación Española de Entomología 44: 203-206.
4. Cabanillas D, Parejo-Pulido D (2019) Primer registro de *Lamyctes* (*Lamyctes*) *emarginatus* (Newport, 1844) (Chilopoda: Lithobiomorpha: Henicopidae) en la Comunidad Autónoma de Extremadura y otras citas de la provincia de Badajoz (España). Boletín de la Sociedad Entomológica Aragonesa 64: 307-311.
5. Cabanillas D (2018) Introducción al conocimiento de los quilópodos. Mundo Artropodo 4: 7-18.
6. Cabanillas D (2019) Ampliación de la distribución de *Scolopendra cingulata* Latreille, 1829 y *Scolopendra oraniensis* Lucas, 1846 (Chilopoda, Scolopendromorpha, Scolopendridae) en la Comunidad de Madrid (España). Boletín de la Asociación española de Entomología 43: 55-77.
7. Cabanillas D, Rodríguez G, Morales-Mata JI, Ortega-Quintanilla, S (2021) A preliminary catalogue of the centipede species (Myriapoda:Chilopoda) of the province of Córdoba (Andalucía, southern Spain). Boletín de la Socieada Andaluza de Entomología 31: 57-70.
8. Carballo J, Daza JL (1991) Contribución al estudio faunístico de la clase Chilopoda en Andalucía occidental. Arquivos do Museu Bocage. Nova Série 2(5): 79-116.
9. Carballo J, Escot C, Estacio F, Pablos F (1988) Contribución al conocimiento de la Clase Chilopoda de Andalucía Occidental II. Actas III Congreso Ibérico de Entomología, Granada 65-72.
10. Carballo J, Escot C, Estacio F, Ortega F, Pablos F (1986) Contribución al conocimiento de los Chilopoda de Andalucía Occidental. (I). Actas de las VIII Jornadas de la Asociación española de Entomología, Sevilla, 1097-1106.
11. Daza JL, Galindo M, Conradi-Barrena M, Coveñas P (1991) Contribucion al conocimiento de la clase Chilopoda de Andalucia Occidental III. Arquivos do Museu Bocage 2(6): 117-125.
12. Doménech C, Cabanillas D, Gilgado JD, Garcia L, Hernández-Corral J (2023) Contribución al conocimiento de los ciempiés, milpiés (Myriapoda: Chilopoda, Diplopoda) e isópodos terrestres (Crustacea: Oniscidea) de la Sierra de Aitana (Alicante, España). Arxius de Miscel·lània Zoològica 21: 129-149.  <https://doi.org/10.32800/amz.2023.21.0129>
13. Doménech C (2016) Introducción al terrario de miriárpodos del género *Scolopendra*. Jangala Magazine 6: 4-14.
14. Foddai D, Minelli A, Zapparoli M (1996): I chilopodi delle isole circumsarde nel contesto del popolamento insulare dell`area tirrenica s.l. Biogeographia 18: 357-376
15. García-Ruíz A, Serra A (2003) Studies on centipede communities (Chilopoda) from three habitats in Toledo Province, Spain. African Invertebrates 44(1): 227-236.
16. García-Ruiz A (1993) Contribución al conocimiento de los quilópodos de la provincia de Jaén: I Puerto de Despeñaperros. Boletin del Grupo Entomologico de Madrid 6: 27-32.
17. García-Ruiz, A (1995) Some cases of structural abnormality in Scolopendra (Chilopoda, Scolopendromorpha). Brithish Bulletin of the British Myriapod Group 11: 33-35.
18. García-Ruiz A (1997) Estudio faunístico y taxonómico de los quilópodos (Myriapoda, Chilopoda) de la provincia de Toledo. Ed. Universidad Complutense, Madrid 342 pp.
19. García-Ruiz A (2007) Estudio de la actividad espacial de *Scolopendra cingulata* y *Scolopendra canidens oraniensis*. Boletín Sociedad Entomológica Aragonesa 40: 569−570.
20. García-Ruiz A (2018) Contribución al conocimiento cavernícola de *Scolopendra canidens, subsp. oraniensis* (Lucas, 1846) (Myriapoda, Chilopoda). Monografías Bioespeleológicas 13: 13-16.
21. Giribet G (2015) Orden Scolopendromorpha. Revista Ibero Diversidad Entomologica (Sociedad Española de Entomología) 30: 1-9.
22. Iorio É, Geoffroy JJ (2006) Contribution à la connaissance de *Scolopendra oraniensis* H. Lucas, 1846 (Chilopoda, Scolopendromorpha, Scolopendridae). Le bulletin d’Arthropoda 27: 48-51.
23. Iorio É, Geoffroy JJ (2008) Les scolopendromorphes de France (Chilopoda, Scolopendromorpha): identification et distribution géographique des espèces. Riviéra scientifique 91: 73-90.
24. Iorio E (2003) Morphologie externe des appareils génitaux mâle et femelle de la famille Scolo­pendridae (Chilopoda, Scolopendromorpha). Bulletin de Phyllie 16: 10-16.
25. Lewis JGE (1985) Possible species isolation mechanisms in some scolopendrid centipedes (Chilopoda; Scolopendridae). Bijdragen tot de Dierkunde 55(1): 125-130.
26. Lewis JGE (2010) A key and annotated list of the *Scolopendra* species of the Old World with a reappraisal of *Arthrorhabdus* (Chilopoda: Scolopendromorpha: Scolopendridae). International Journal of Myriapodology 3: 83–122. <https://doi.org/10.1163/187525410X12578602960380>
27. Minelli A (1983) On Sardinian centipedes. Bollettino di Zoologia 49: 1-16.
28. Minelli A, Spolaor A, Tortani S (1978) I chilopodi delle Isole Eolie. Animalia 8: 37-42.
29. Negrea S, Matic, Z (1973) Chilopodes cavernicoles et endogés de l’île de Majorque. Mission biospéologique Constantin Dragan (1970-1971). Bolletí de la Societat d’Història Natural de les Balears 18: 21-39.
30. Ortuño VM, Gilgado JD, Jiménez-Valverde A, Sendra A, Pérez-Suárez G, Herrero-Borgoñón JJ (2018) The "Alluvial Mesovoid Shallow Substratum", a new subterranean habitat. PLoS One, 4;8(10): e76311. http://doi: 10.1371/journal.pone.0076311.
31. Sammler S, Voigtländer K, Stoev P, Enghoff H, Müller CHG (2006) New studies on myriapods (Chilopoda, Diplopoda) from Ibiza with a checklist for the Balearic Islands. Norwegian Journal of Entomology 53: 299-309.
32. Santibañez FJ, Garcia-Ruiz A (1995) Estudio de una comunidad de quilópodos (Myriapoda, Chilopoda) en un campo de cultivo  abandonado. Avances  en  Entomología ibérica 159-164.
33. Serra A (1983) Els Scolopendrinae i els Theatopsinae (Chilopoda. Scolopendromorpha) de la Península Ibérica. Butlletí de la Institutició Catalana d’Historia Natural 49(5): 77-83.
34. Serra A (1985) Contribución al conocimiento de los Scolopendromorpha del sur de la Península Ibérica. Publicaciones del Departamento de Zoologia 11: 37-43.
35. [Simaiakis S, Mylonas M (2008) The *Scolopendra* species (Chilopoda: Scolopendromorpha: Scolopendridae) of Greece (E-Mediterranean): a theoretical approach on the effect of geography and palaeogeography on their distribution. Zootaxa 1792: 39-53](https://tb.plazi.org/GgServer/summary/FFB1FFA02714892DBB0D3A5EFFFCFFB3).
36. [Simaiakis SM, Edgecombe GD (2013) Scolopendromorph centipedes (Chilopoda: Scolopendromorpha) in the Natural History Museum (London): A review of the hitherto unidentified species collected in Africa, with remarks on taxonomy and distribution, and a new species of *Otostigmus* (*Parotostigmus*) Zootaxa 3734(2): 169-198](https://tb.plazi.org/GgServer/summary/FF8FFC20FFB0FFF7FFC1AA2DFF95FFA1).
37. Vadell M, Martínez M (2011) Sobre algunos quilópodos de Son Real (Santa Margarita, Mallorca). Bolletí de la Societat d’Història Natural de les Balears 54: 75-84.
38. Vadell M (2010) *Scolopendra oraniensis*. In: *BioAtles*. Ed. Govern de les Illes Balears, Palma 5 edició.
39. Voigtländer K, Reip HS (2013) Morphological, taxonomical and ecological contributions to the chilopod fauna of Andalusia (Sierra de Grazalema and Los Alcornocales), Spain. Graellsia 69(2): 217-241.
40. Würmli M (1980) Statistische Unterschungen zur Systematik und postembryonalen Entwicklung der Scolopendra canidens Gruppe (Chilopoda: Scolopendromorpha). Sitzungsberichte der Österreichischen Akademie der Wissenschaften 189: 315-353.
41. [Zapparoli M, Iorio E (2012) The centipedes (Chilopoda) of Corsica: catalogue of species with faunistic, zoogeographical and ecological remarks. International Journal of Myriapodology 7: 15-68](https://tb.plazi.org/GgServer/summary/FFE29A79FFC9C96B3916FFF4FFE9FFE7).
42. Zapparoli M (1986) Osservazioni sui Chilopodi dell'Appennino lucano e calabrese (Chilopoda). Biogeographia 10: 311-340.
43. Zapparoli M (2009) An annotated catalogue of the epigeic and cave centipedes (Chilopoda) of Sardinia. Zootaxa 2318: 56-168.

| **Supplementary File 3.** *Scolopendra chlorotes* neotype (= *S. oraniensis*) and “topotype” morphological comparison. AP, apical spines. SAP, subapical spines. DS, dorsal spines. LS, lateral spines. VL, ventro-lateral spines. V, ventral spines. VM, ventro-median spines. M, median spines. DM, dorso-median spines. SP, prefemoral process spines. UL, ultimate legs. ULBS, ultimate leg-bearing segment. T, tergite. TT, tergites. S, sternite. SS, sternites. RG, Retracted genitalia. SN, Supernumerary spines between VM and M rows. *= Not visible, damaged, or regenerated. | | | | | | | | |
| --- | --- | --- | --- | --- | --- | --- | --- | --- |
|  | **“Topotype 1” CEUAMr25** | **“Topotype 2” CEUAMr26** | **“Topotype 3” CEUAMr27** | **“Topotype 4” CEUAMr28** | **Neotype**  **“Topotype 5” CEUAMr29** | **“Topotype 6” CEUAMr30** | **“Topotype 7” CEUAMr31** | **“Topotype 8” CEUAMr32** |
| **Locality** | Málaga | Málaga | Málaga | Málaga | Alahurín de la Torre | Alahurín de la Torre | Casabermeja | Casabermeja |
| **Body length in mm** | 25 | 38 | 45 | 40 | 48 | 43 | 64 | 46 |
| **Sex** | Female | Female | Female | Female | Male, penis retracted | Female | Female | Female |
| **Compatible colouration with the original description** | Yes | Yes, tegument yellowish | Yes, tegument yellowish | Yes | Yes | Yes | Yes, tegument yellowish | Yes, tegument yellowish |
| **Antenna reaching to tergite** | T3 | T3 | T3 | T3 | T3 | T3 | T3 | T3 |
| **Number of antennal articles** | 19/19 | 19/19 | 20/19 | 19/20 | 19/19 | 19/19 | 10*/19 | 19/18 |
| **Number of proximal glabrous articles** | 5½ | 5½ | 5 | 5½ | 5½ | 5½ | 5½ | 5_1/3_ |
| **Teeth on tooth plate** | 2*+4 | 3+3 | 3+3 | 4+4 | 3+3 | 4+4 | 4+4 | 4+4 |
| **Teeth on forcipular trochanteroprefemoral processes**  **Total (apical /medial)** | 3(1/2) - 3(1/2) | 3(2/1) - 3(2/1) | 2(1/1) - 2(1/1) | 3(1/2) - 3(1/2) | 3(1/2) - 2(1/1) | 3(1/2) - 3(1/2) | 3(1/2) - 3(1/2) | 3(1/2) - 3(1/2) |
| **Tergite paramedian sutures** | TT2-20 | TT2-20 | TT2-20 | TT2-20 | TT2-20 | TT2-20 | TT2-20 | TT2-20 |
| **Longitudinal suture on T21** | Present | Present | Present | Present | Present | Present | Present | Present |
| **First tergite with complete margination** | 17 | 18 | 18 | 17 | 14 | 16 | 17 | 17 |
| **Paramedian sutures on sternites** | SS2-20 | SS2-20 | SS2-20 | SS2-20 | SS2-20 | SS2-20 | SS2-20 | SS2-20 |
| **Spines in coxopleuron**  **(Left/Right)** | 1/1 | 1/1 | 1/1 | 1/1 | 1/1 | 1/1 | 1/1 | 1/1 |
| **Spines in coxopleural process (Left/Right)** | AS-SAP: 4/3  DS: 1/1  LS: 3/3  Total:8/7 | AS-SAP: 5/5  DS: 1/1  LS: 2/3  Total:8/9 | AS-SAP: 5/4  DS: 4/1  LS: 2/1  Total:11/6 | AS-SAP: 6/5  DS: 2/4  LS: 2/2  Total:10/11 | AS-SAP: 5/5  DS: 3/2  LS: 3/2  Total:10/10 | AS-SAP: 5/5  DS: 3/4  LS: 2/2  Total:10/11 | AS-SAP: 5/4  DS: 1/1  LS: 3/3  Total:9/9 | AS-SAP: 5/5  DS: 2/2  LS: 3/2  Total:10/9 |
| **Tarsal spurs on leg 1 (Left/Right)** | 2/2 | 2/2 | 2/1 | 2/2 | 2/2 | 2/2 | 2/2 | 1/2 |
| **Legs with one tarsal spur** | 2-19 | 2-19 | 2-19 | 2-19 | 2-19 | 2-19 | 2-19 | 2-19 |
| **Ultimate legs prefemoral spinulation and spines in prefemoral process (Left/Right)** | VL: 6/9  V: 6/6  SN: 0/3  VM: 6/6  M: 6/6  DM: 3/2  SP: 1/2  Total: 28/34 | VL: 6/5  V: 4/5  VM: 3/7  M: 7/10  DM: 2/2  SP: 2/2  Total: 24/31 | VL: 7/7  V: 5/ 2  SN: 2/0  VM: 3/3  M: 3/10  DM: 2/3  SP: 2/3  Total: 22/32 | VL: 7/6  V: 6/7  VM: 5/2  M: 9/6  DM: 2/3  SP: 2/2  Total: 31/26 | VL: 2/7  V: 5/6  VM: 6/7  M: 5/6  DM: 3/2  SP: 2/2  Total: 23/30 | VL: 5/5  V: 6/6  VM: 6/6  M: 8/7  DM: 2/2  SP: 2/2  Total: 29/28 | VL: 10*/5  V: 8*/5  VM: 4*/7  M: 6*/5  DM: 7*/1  SP: 5*/0  Total: 40/23 | VL: 5/8  V: 6/5  SN: 2/1  VM: 5/4  M: 4/2  DM: 4/4  SP: 3/2  Total: 28/26 |

| **Supplementary File 3 (continuation).** *Scolopendra chlorotes* neotype (= *S. oraniensis*) and “topotypes” morphological comparison. AP, apical spines. SAP, subapical spines. DS, dorsal spines. LS, lateral spines. VL, ventro-lateral spines. V, ventral spines. VM, ventro-median spines. M, median spines. DM, dorso-median spines. SP, prefemoral process spines. UL, ultimate legs. ULBS, ultimate leg-bearing segment. T, tergite. TT, tergites. S, sternite. SS, sternites. RG, Retracted genitalia. SN, Supernumerary spines between VM and M rows. *= Not visible, damaged, or regenerated. | | | | | | | | |
| --- | --- | --- | --- | --- | --- | --- | --- | --- |
|  | **“Topotype 9” CEUAMr33** | **2Topotype 10” CEUAMr34** | **“Topotype 11” CEUAMr35** | **“Topotype 12” CEUAMr36** | **“Topotype 13” CEUAMr37** | **“Topotype 14” CEUAMr38** | **“Topotype 15” CEUAMr39** | ***S. chlorotes***  ***“*Topotypes*”***  **(CEUAMr26-40)** |
| **Locality** | Casabermeja | Casabermeja | Totalán | Totalán | Totalán | Estepona | Estepona | - |
| **Body length in mm** | 47 | 33 | 36 | 31 | 26 | 39 | 35 | 26-64 |
| **Sex** | Male* | RG | Male | Male | RG | Female; partially retracted | RG | - |
| **Compatible colouration with the original description** | Yes, tegument yellowish | Yes, tegument yellowish | Yes, tegument yellowish | Yes, tegument yellowish | Yes, tegument dark green | Yes, tegument dark green | Yes, tegument dark green | Yes |
| **Antenna reaching to tergite** | T3 | T3 | T3 | T3 | T3 | T3 | T3 | T3 |
| **Number of antennal articles** | 19/19 | 19/19 | 18/19 | 19/19 | 19/19 | 19/17 | 16*/20 | 17-20 |
| **Number of proximal glabrous articles** | 5_1/3_ | 5 | 5_1/3_ | 5½ | 5 | 5½ | 5 | 5-5½ |
| **Teeth on tooth plate** | 4+4 | 4+4 | 4+4 | 4+4 | 3+3 | 3+3 | 3+3 | 3+3; 4+4 |
| **Teeth on forcipular trochanteroprefemoral processes**  **Total (apical /medial)** | 3(1/2) - 3(1/2) | 3(1/2) - 3(1/2) | 3(1/2) - 3(1/2) | 3(1/2) - 3(1/2) | 2(1/1) - 3(1/2) | 2 (1/1) - 2 (1/1) | 2 (1/1) - 2 (1/1) | 2(1/1); 3(1/2) |
| **Tergite paramedian sutures** | TT2-20 | TT2-20 | TT2-20 | TT2-20 | TT2-20 | TT2-20 | TT2-20 | TT2-20 |
| **Longitudinal suture on T21** | Present | Present | Present | Present | Present | Present | Present | Present |
| **First tergite with complete margination** | 15 | 17 | 15 | 17 | 17 | 17 | 18 | 14-18 |
| **Paramedian sutures on sternites** | SS2-20 | SS2-20 | SS2-20 | SS2-20 | SS2-20 | SS2-20 | SS2-20 | SS2-20 |
| **Spines in coxopleuron**  **(Left/Right)** | 1/1 | 1/1 | 1/1 | 1/1 | 1/1 | 1/1 | 1/1 | 1/1 |
| **Spines in coxopleural process (Left/Right)** | AS-SAP: 5/5  DS: 1/0  LS: 3/4  Total:9/9 | AS-SAP: 5/5  DS: 2/2  LS: 3/3  Total:10/10 | AS-SAP: 7/4  DS: 2/2  LS: 3/4  Total:12/10 | AS-SAP: 5/5  DS: 3/3  LS: 3/2  Total:10/10 | AS-SAP: 7/5  DS: 2/2  LS: 1/2  Total:10/9 | AS-SAP: 6/5  DS: 1/2  LS: 3/3  Total:11/10 | AS-SAP: 4/5  DS: 3/2  LS: 3/3  Total:10/10 | AP-SAP: 4-7  DS: 0-4  LS: 1-4  Total:5-15 |
| **Tarsal spurs on leg 1 (Left/Right)** | 2/2 | 2/2 | 2/2 | 2/* | 2/2 | 2/2 | 2/2 | 1-2 |
| **Legs with one tarsal spur** | 2-19 | 2-19 | 2-19 | 2-19 | 2-19 | 2-19 | 2-18 | 2-18 or 19 |
| **Ultimate legs prefemoral spinulation and spines in prefemoral process (Left/Right)** | VL: 5/8  V: 4/6  VM: 5/5  M: 4/4  DM: 2/3  SP: 2/1  Total: 22/27 | VL: 6/6  V: 6/10  VM: 4/8  M: 3/3  DM: 4/2  SP: 2/2  Total: 25/32 | VL: 6/*12  V: 5/*5  SN: 0/3  VM: 6/*8  M: 8/*11  DM: 5/*4  SP: 2/* 5  Total: 32/*48 | VL: 6/6  V: 5/5  VM: 7/4  M: 8/6  DM: 2/2  SP: 2/2  Total: 30/25 | VL: 5/7  V: 6/4  VM: 6/5  M: 7/7  DM: 3/3  SP: 2/2  Total: 29/28 | VL: 7/8  V: 6/6  VM: 4/7  M: 7/6  DM: 2/2  SP: 2/2  Total: 28/31 | VL: 6/8  V: 6/6  VM: 6/7  M: 7/6  DM: 2/2  SP: 2/2  Total: 29/31 | VL: 2-10  V: 4-10  VM: 2-8  M: 2-10  DM: 1-7  SP: 0-5  Total: 11-50 |
